# Haptotropism in a Nickel Complex with a Neutral, π‐Bridging *cyclo*‐P_4_ Ligand Analogous to Cyclobutadiene

**DOI:** 10.1002/anie.202115692

**Published:** 2022-03-09

**Authors:** Chris Gendy, Juuso Valjus, Heikki M. Tuononen, Roland Roesler

**Affiliations:** ^1^ Department of Chemistry University of Calgary 2500 University Drive NW Calgary AB, T2N 1N4 Canada; ^2^ Department of Chemistry Nanoscience Centre University of Jyväskylä P.O. Box 35 40014 Jyväskylä Finland

**Keywords:** Haptotropism, *N*-Heterocyclic Carbene, Nickel, Phosphorus, π-Ligands

## Abstract

Dedicated to Professor Manfred Scheer on the occasion of his 65th birthday

The reaction of (**1**)Ni(η^2^‐cod), **2**, incorporating a chelating bis(*N*‐heterocyclic carbene) **1**, with P_4_ in pentane yielded the dinuclear complex [(**2**)Ni]_2_(μ_2_,η^2^ : η^2^‐P_4_), **3**, formally featuring a cyclobutadiene‐like, neutral, rectangular, π‐bridging P_4_‐ring. In toluene, the butterfly‐shaped complex [(**1**)Ni]_2_(μ_2_,η^2^ : η^2^‐P_2_), **4**, with a formally neutral P_2_‐unit was obtained from **2** and either P_4_ or **3**. Computational studies showed that a haptotropic rearrangement involving two isomers of the μ_2_,η^2^ : η^2^‐P_4_ coordination mode and a low‐energy μ_2_,η^4^ : η^4^‐P_4_ coordination mode, as previously predicted for related nickel cyclobutadiene complexes, could explain the coalescence observed in the low‐temperature NMR spectra of **3**. The insertion of the (**1**)Ni fragment into a P−P bond of P_7_(SiMe_3_)_3_, forming complex **5** with a norbornane‐like P_7_ ligand, was also observed.

## Graphical abstract

The solvent‐dependent reaction of white phosphorus with a Ni^0^ reagent incorporating a chelating bis(*N*‐heterocyclic carbene) leads to complexes with neutral P_2_ and P_4_ ligands. The latter mirrors the coordination of cyclobutadiene to Ni^0^, displaying olefin‐like bonding and η^2^‐η^4^‐haptotropism, and can be converted to the former. These findings emphasize the fluidity of *cyclo*‐P_4_ coordination modes.

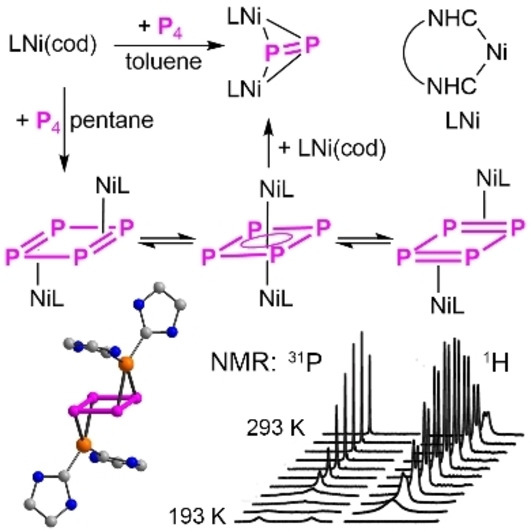

Transition metal complexes incorporating P_*x*_ ligands have been extensively investigated due to their appealing structural variety and intriguing bonding.^[1]^ White phosphorus is the entryway to the production of most phosphorus compounds,^[2]^ and more recently the interest toward P_*x*_ metal complexes has expanded to include the metal‐mediated activation and further transformation of this molecule.^[3]^ Complexes incorporating P_4_ ligands are of particular interest because they are hypothesized to constitute the first stage in the activation of P_4_.^[4]^ Hydrocarbon‐based π‐ligands such as cyclobutadiene,^[5]^ cyclopentadienyl, and benzene have been used as a guideline for systematizing the chemistry of substituent‐free, or “naked”, phosphorus ligands because the CH and P fragments are isolobal.^[6]^

Mirroring cyclobutadiene complexes **A** (Figure 1), the planar *cyclo*‐P_4_ ligand forms mononuclear, 18‐valence‐electron sandwich and half sandwich complexes **B** (M=V,^[7]^ Nb,^[8]^ Ta,^[9]^ L_*n*_=Cp^x^(CO)_2_; M=Mo,^[10]^ L_*n*_=(CO)_2_(CNR)_2_; (CO)I_2_(CNR)_2_; M=Fe,^[11]^ L_*n*_=(Cy_2_PCH_2_CH_2_)_2_PPh; M=Co,^[12]^ L_*n*_=Cp^x^), **B** ^−^ (M=Mo,^[13]^ L_*n*_=(CO)I(CNR)_2_; M=Fe,^[14]^ L_*n*_=Cp^x^; M=Co,^[15]^ L =bis(2,6‐Dipp)phenanthrene‐9,10‐diimine), and **B** ^2−^ (M=Mo,^[13]^ L_*n*_=(CO)(CNR)_2_, (CO)_2_(CNR)). The phosphorus ring in **B** can σ‐donate up to four lone pairs to additional metals, generating multinuclear complexes.^[1]^ By analogy to cyclobutadiene, the P_4_ ligand in **B** is considered to be dianionic, and this view is supported by Mössbauer measurements^[11]^ and computational studies.[^13^, ^14^] *cyclo*‐P_4_^2−^ has been described as antiaromatic as a free ligand,^[16]^ lone pair aromatic in alkali metal salts,^[17]^ and aromatic in transition metal complexes.[^11^, ^13^]

**Figure 1:**
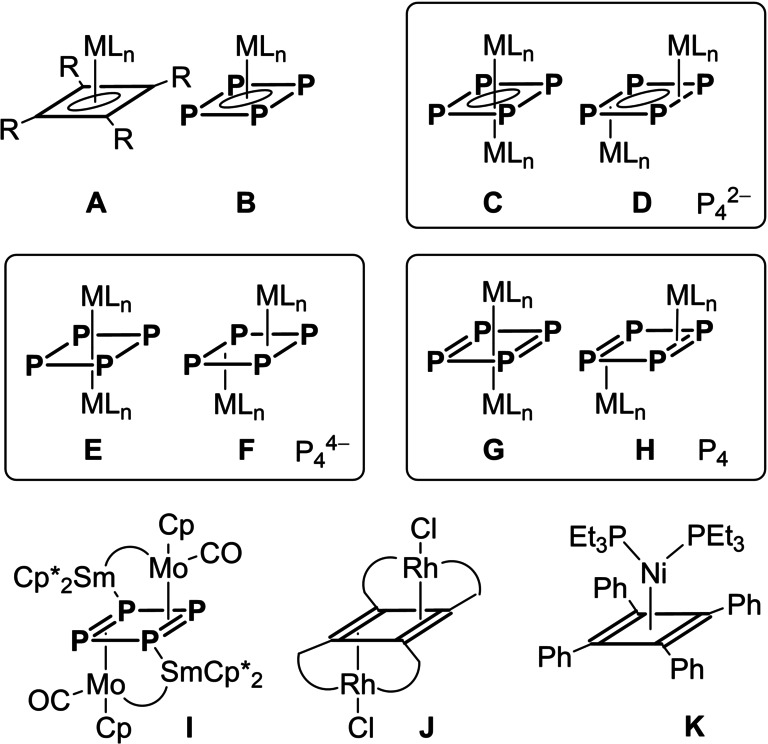
Reported coordination modes of the planar cyclo‐P_4_ ligand (**B**–**I**) and relevant cyclobutadiene analogues (**A**, **J**, and **K**).

In contrast to cyclobutadiene, which rarely π‐bridges transition metals,^[18]^ *cyclo*‐P_4_ forms a variety of bridged complexes where the planar ligand can be formally considered P_4_^2−^ (**C** and **D**), P_4_^4−^ (**E** and **F**), or P_4_ (**G** and **H**), based on structural, spectroscopic, and computational data. For example, the μ_2_ : η^4^,η^4^‐bridging mode has been observed in complexes of type **C** (M=Fe,^[19]^ L_*n*_=β‐diketiminato; M=Sm,^[20]^ L_*n*_={[DippN]_2_CH}_2_), **C** ^−^ (M=Co, L_*n*_=β‐diketiminato),[^21^, ^22^] **E** (M=Zr, L_*n*_=PhP(CH_2_SiMe_2_NSiMe_2_CH_2_)_2_PPh),^[23]^ **E** ^−^ (M=Co, L_*n*_=BIAN)^[24]^ and **G** (M=Co, L_*n*_=β‐diketiminato),[^21^, ^22^] while complexes **D** (M=U, L_*n*_=Cp*(COT))^[25]^ and **F** (M=Nb,^[26]^ L_*n*_=(DippO)_3_; M=Nb, Ta,^[27]^ L=(β‐diketiminato)(*t*BuN)) display μ_2_ : η^2^,η^2^‐ and μ_2_ : η^3^,η^3^‐bridging modes, respectively. Complex **I** is the only known example featuring a neutral μ_2_ : η^2^,η^2^‐P_4_ ligand, presumably because the constrained geometry does not allow for η^4^‐coordination.^[28]^ This mirrors the behaviour of cyclobutadiene, which is known to coordinate in μ_2_ : η^2^,η^2^‐fashion only in **J**.^[29]^

Although the chemistry of nickel with P_4_ has been extensively investigated, leading to the isolation of a variety of (L_*n*_Ni)_y_P_*x*_ derivatives (*x*=2–5, 8),[^1^, ^3^, ^30^] a planar, π‐coordinating *cyclo*‐P_4_ nickel complex has remained elusive.^[31]^ The excellent π‐donating properties of the metal in [(**1**)Ni(η^2^‐cod)],^[32a]^ **2** (Scheme 1; **1**=bis(NHC)),^[32b]^ recommend it as a promising synthon for the stabilization of π‐bonded P_*x*_‐ligands. In a similar manner, the (Et_3_P)_2_Ni fragment has allowed the isolation of complex **K**, having structural features consistent with Ni^0^ and a neutral, η^4^‐cyclobutadiene ligand, as opposite to Ni^II^ and cyclobutadienyl.^[33]^ Furthermore, a computational study on the model system (cyclobutadiene) Ni(PH_3_)_2_ suggested that the η^4^‐ and η^2^‐coordination modes had similar energies and a tiny interconversion barrier.^[34]^ Higher‐level calculations for the analogous (cyclobutadiene)Pt(diphosphinylethane) gave a slightly greater energy difference.^[35]^ These findings led us to investigate the reactivity of **2** with P_4_ in pursuit of the elusive μ_2_ : η^2^,η^2^‐P_4_ bridging mode **H**. This chemistry, as well as the reactivity of **2** with P_7_(SiMe_3_)_3_, will be reported herein.

**Scheme 1:**
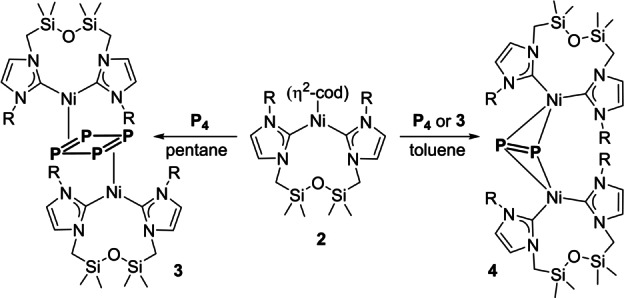
Synthesis of **3** and **4**.

Reaction of **2** with P_4_ in pentane afforded diamagnetic, crystalline complex [(**1**)Ni]_2_P_4_, **3**, irrespective of stoichiometry (Scheme 1). An X‐ray diffraction experiment revealed a dinuclear, *C*_i_‐symmetric structure with a planar, rectangular μ_2_ : η^2^,η^2^‐P_4_ core (Figure 2). The *cyclo*‐P_4_ ligand features alternating long (2.242(8) Å) and short (2.149(3) Å) P−P bonds (cf. P−P 2.1994(3) Å in P_4_ ^[36]^ and P=P 2.140(1) in PhP=PPh coordinated to Ni^0^),^[37]^ suggestive of a neutral P_4_ ligand bound by two (**1**)Ni^0^ fragments, i.e., **H**. For comparison, the P_4_ ligand adopts a very similar geometry in **G**, M=Co^I^ (*av*. P−P 2.29 Å and P=P 2.13 Å).[^21^, ^22^]

**Figure 2:**
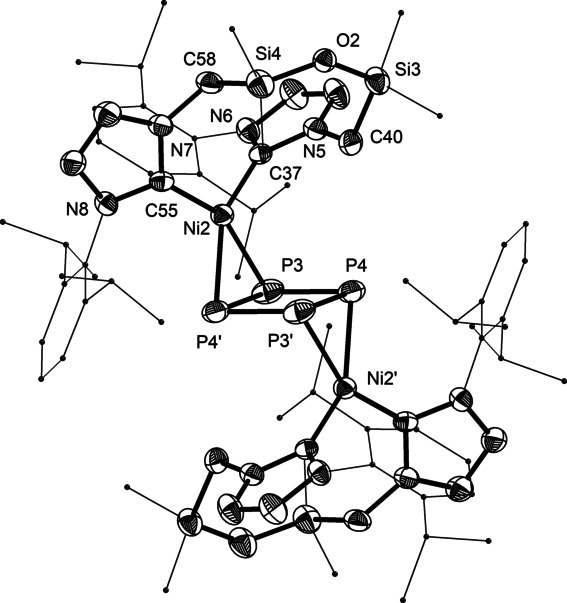
Solid‐state structure of one of the two independent molecules of **3**, with 50 % thermal ellipsoids and hydrogen atoms omitted for clarity. Selected bond lengths [Å] and angles [°]: P3–P4′ 2.145(2), 2.149(2), P3–P4 2.237(2), 2.242(2), Ni–P 2.2436(15)–2.2822(14), Ni–C 1.911(5)–1.933(5); P‐P‐P 89.58(7)–90.42(7), C‐Ni‐C 109.9(2), 110.8(2).^[38]^

A room‐temperature ^31^P NMR spectrum of **3** in toluene‐*d*_8_ revealed a single, broad resonance at 45 ppm (cf. 128 and 169 ppm for **G**, M=Co^I^, in the solid state),^[21]^ indicative of a dynamic process. The variable‐temperature study revealed two coalescence temperatures (Figure 3). At low temperature, the presence of two ^31^P resonances (21 and 63 ppm) is consistent with a centrosymmetric *C*_i_‐structure, as observed in the solid state. Upon heating above 210 K, all phosphorus atoms become equivalent on the NMR time scale. Resonance broadening suggestive of a second coalescence is apparent slightly above room temperature, but incipient decomposition precluded the investigation of this process. The ^1^H NMR spectrum features four AB doublets for the methylene protons (3.3, 4.3, 5.0, and 8.2 ppm, Figure S1), and two resonances for the carbene carbons are observed in ^13^C NMR (192.8 and 202.7 ppm, Figure S2). This indicates that the solid‐state structure of the (**1**)Ni fragment is retained in solution between 210–293 K.

**Figure 3:**
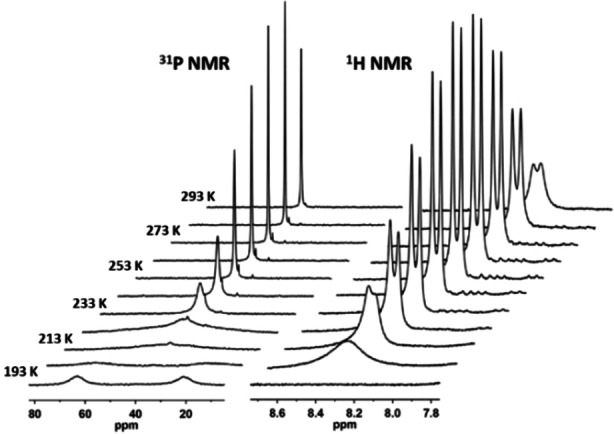
Stack plots of variable‐temperature (193–293 K) ^31^P (left) and ^1^H (right) NMR spectra of **3** in toluene‐*d*_8_.

DFT calculations on **3** yielded a closed‐shell singlet ground state free of instabilities, supporting the proposed coordination mode **H**. An ETS‐NOCV analysis of bonding in **3** using closed‐ shell fragments ((**1**)Ni)_2_ and P_4_ showed significant interaction of nickel *d*‐orbitals with the π‐type orbitals of P_4_, giving two strongly stabilizing contributions (Figure S24). Population analyses suggested a net flow of 0.7–0.8 e^−^ from the two (**1**)Ni^0^ fragments to P_4_, underlining the fact that the use of integer oxidation states and charges is inevitably an oversimplification.

Further inspection of the potential energy surface of **3** with DFT revealed that a μ_2_ : η^4^,η^4^‐bound *C*_i_‐isomer (**3′′**) with a square‐like *cyclo*‐P_4_ ring is only 14 kJ mol^−1^ higher in energy than **3**, while a second *C*_i_‐symmetric μ_2_ : η^2^,η^2^‐bound isomer (**3′**) has a relative energy of only 8 kJ mol^−1^ (Scheme 2, Figure S23). Both **3** and **3′** connect to **3′′** with barriers <50 kJ mol^−1^, in agreement with the value of 44 kJ mol^−1^ calculated from the coalescence temperature. These results are in accordance with a haptotropic rearrangement (P_4_‐ring whizzing) accounting for the low temperature coalescence in the ^31^P NMR data of **3**. Fluxional P_4_‐ring behaviour has been proposed for *cyclo*‐P_4_ complexes.[^11^, ^23^, ^26^] The high‐temperature coalescence is tentatively assigned to ligand symmetrisation via dynamic motion that was shown to involve an activation energy of 53 kJ mol^−1^ in (**1**)NiGeCl_2_;^[32b]^ the barrier for **3** is expected to be higher due to increased steric strain.

**Scheme 2:**
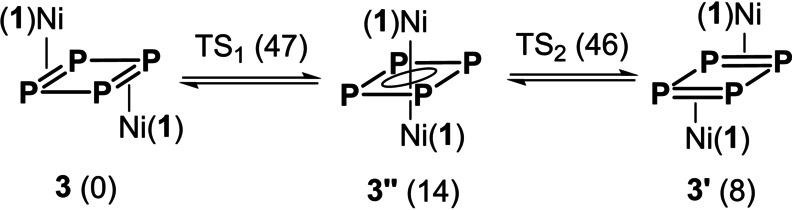
Calculated haptotropic rearrangement between **3** and **3′** (which differ in the relative orientation of ligand **1** with respect to *cyclo‐*P_4_), via **3′′** over transition states TS_1_ and TS_2_. Relative Gibbs free energies (in kJ mol^−1^ at 298 K) in parenthesis.

The singlet ground state of **3′′** shows an instability, leading to a broken‐symmetry singlet solution in which approximately 0.6 α spin becomes localized on one Ni centre and 0.6 β spin on the other. This suggests that coordination mode **C**, involving two (**1**)Ni^I^ fragments bridged by a dianionic P_4_^2−^ ring, is a reasonable first‐order approximation of bonding in the intermediate **3′′**. This description is well in line with the square‐shaped structure calculated for the *cyclo*‐P_4_ ring in **3′′** having two almost equal bond lengths (2.163 and 2.172 Å) and bond angles (88 and 92°).

In toluene, the reaction of **2** with either a stoichiometric or an excess amount of P_4_ yields exclusively **4** (Scheme 1), which can also be prepared from **3** and **2** in toluene; notably, **3** does not convert to **4** upon dissolution in toluene. Under an inert atmosphere at −40 °C, solids **3** and **4** are stable for months but at room temperature in solution decomposition of both compounds leads within hours to the disappearance of all ^31^P resonances and formation of unidentified products. The solid state structure of **4** (Figure S21) features a butterfly‐shaped Ni_2_P_2_ core with a very short (2.0784(16) Å) P−P bond and a dihedral angle of 120.87(3)°. In similar complexes [{(NHC)_2_Ni}_2_(μ,η^2^ : η^2^‐P_2_)], the P_2_ unit (P−P 2.0906(8) Å) was described as P_2_^4−^ based on the visual inspection of frontier orbitals and their localization on P_2_.^[39]^ Quantitative bonding analyses performed for **4** show that, while its frontier Kohn–Sham orbitals are similar to those of [{(NHC)_2_Ni}_2_(μ,η^2^ : η^2^‐P_2_)] (Figure S25), the contribution from P_2_ is much less than 50 % in all cases. Thus, complex **4** can be formally described as a neutral P_2_ ligand bound by two (**1**)Ni(0) fragments, akin to the reported dinuclear Ni^0^‐alkyne complexes.^[40]^ An ETS‐NOCV analysis of **4** using this fragmentation scheme shows charge flow between the *d*‐orbitals on the metals and the π‐type orbitals on P_2_, resulting in both metal‐to‐ligand and ligand‐to‐metal bonding contributions (Figure S26). Population analyses indicate a net flow of 0.5–0.6 e^−^ from ((**1**)Ni)_2_ to P_2_, illustrating, again, that the discussed promolecular fragments should only be treated as good first‐order approximations of bonding in **3** and **4**.

The most accessible phosphorus clusters other than P_4_ are P_7_R_3_, which were used extensively as a ligands;^[41]^ P−P bond activation has only been reported for the parent Zintl ion P_7_^3−^.^[42]^ Reaction of **2** with P_7_(SiMe_3_)_3_ ^[43]^ resulted into the formation of **5** (Scheme 3). At room temperature, short reaction times were needed to limit thermal decomposition. Crystallographic analysis revealed that the (**1**)Ni fragment inserted into the P_3_ ring of P_7_(SiMe_3_)_3_, generating a complex with a norbornane‐like P_7_ ligand (Figure 4). This mirrors the transformations observed for the activation of P_7_^3−^,^[44]^ except in the latter case η^4^‐P_7_^3−^ complexes were usually obtained. The insertion was accompanied by an inversion at phosphorus, leading to a change of the P_7_(SiMe_3_)_3_ conformation from *syn*, which is typical to all P_7_R_3_ analogues, to *anti*. The Ni−P bonds in **5** measure 2.2253(16) and 2.2950(16) Å, while the P−P bonds change little in comparison to P_7_(SiMe_3_)_3_.^[45]^ The ^31^P NMR spectrum of **5** in toluene‐*d*_8_ features seven multiplet signals that were assigned using 2D NMR.

**Scheme 3:**
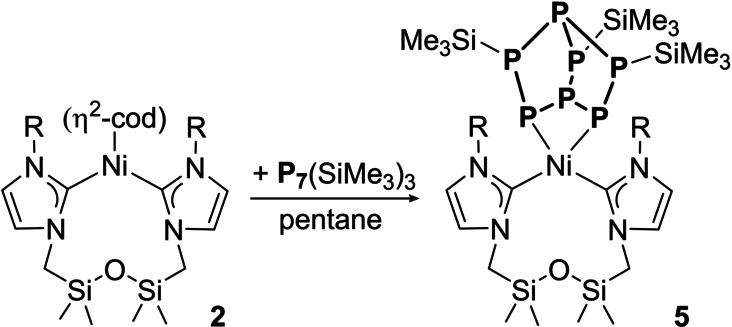
Synthesis of **5**.

**Figure 4:**
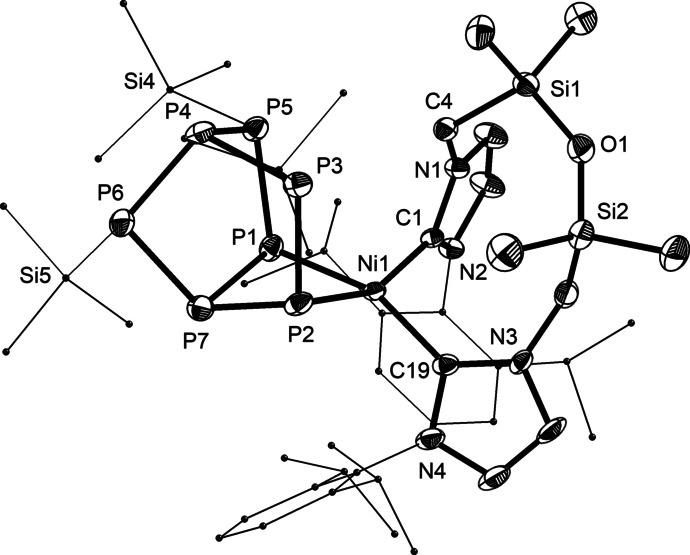
Solid‐state structure of **5** with 50 % thermal ellipsoids and hydrogen atoms omitted for clarity. Selected bond lengths [Å]: P4–P3 2.184(2), P4–P5 2.186(2), P4–P6 2.201(2), P1–P5 2.227(2), P2–P3 2.233(2), P7–P6 2.160(2), P1–P7 2.205(2), P2–P7 2.220(2), P1⋅⋅⋅P2 3.007(2), Ni–C 1.954(5), 1.992(5), Ni1–P1 2.2253(16), Ni1–P2 2.2950(16).^[38]^

In conclusion, complex **2** displays solvent‐dependent reactivity with P_4_, leading to **3** in pentane and **4** in toluene. Moreover, in reaction with **2**, **3** generates **4**, supporting the postulate that *cyclo*‐P_4_ complexes represent the first step in the activation of P_4_ by transition metals. Compound **3** features the elusive μ_2_ : η^2^,η^2^‐P_4_ bridging mode **H**, previously only characterized in the geometry‐constrained system **I**. The low‐temperature coalescence observed in the NMR spectra of **3** can be explained with a haptotropic rearrangement involving two isomers of the crystallographically characterized μ_2_ : η^2^,η^2^ bridging mode, as well as a slightly less stable isomer with a μ_2_ : η^4^,η^4^‐bound ligand. This leads to the equivalence of all phosphorus atoms of **3** on the NMR timescale and mirrors the ring‐whizzing mechanism proposed for the analogous nickel‐cyclobutadiene complex **K**. Employing **2**, the first P−P bond activation in a P_7_R_3_ cluster was also achieved, leading to complex **5**. The low energy barrier haptotropism of **3** and the formalism that best describes the structures **3**, **3′**, and **3′′** suggest that the classification of *cyclo*‐P_4_ complexes in categories **C**–**H** (Figure 1) is somewhat fluid.

## Conflict of interest

The authors declare no conflicts of interest.

## Supporting information

As a service to our authors and readers, this journal provides supporting information supplied by the authors. Such materials are peer reviewed and may be re‐organized for online delivery, but are not copy‐edited or typeset. Technical support issues arising from supporting information (other than missing files) should be addressed to the authors.

Supporting Information

Supporting Information

Supporting Information

Supporting Information
