# Supplementary material for: Haptotropism in a Nickel Complex with a Neutral, π‐Bridging cyclo‐P4 Ligand Analogous to Cyclobutadiene
Source: Angew Chem Int Ed Engl. 2022 Mar 9;61(18):e202115692. doi: 10.1002/anie.202115692 (PMC9313578; doi:10.1002/anie.202115692)
Supplement: Supplementary file 4 — Supporting Information [file ANIE-61-0-s003.pdf]

Angewandte Chemie
Eine Zeitschrift der Gesellschaft Deutscher Chemiker

Supporting Information

Haptotropism in a Nickel Complex with a Neutral, π-Bridging *cyclo*-P_4 Ligand Analogous to Cyclobutadiene

C. Gendy, J. Valjus, H. M. Tuononen, R. Roesler**

Supporting Information

Table of Contents

Synthetic Details 3

General Considerations.......... 3

Synthesis of {[(1)Ni]$_2$(μ$_2$:η^2,η^2-P$_4$)}, 3.......... 3

Synthesis of {[(1)Ni]$_2$(μ$_2$:η^2,η^2-P$_2$)}, 4.......... 4

Synthesis of {[(1)Ni]P$_7$(SiMe$_3$)$_3$}, 5.......... 6

NMR spectra 8

Figure S1. ^{1}H NMR spectrum of 3 (400 MHz, toluene-d_8, 253 K).......... 8

Figure S2. ^{13}C DEPTQ NMR spectrum of 3 (400 MHz, toluene-d_8, 253 K).......... 9

Figure S3. ^{31}P NMR spectrum of 3 (400 MHz, toluene-d_8, 293 K).......... 10

Figure S4. ^{1}H–^{13}C HSQC NMR spectrum of 3 (400 MHz, toluene-d_8, 253 K).......... 11

Figure S5. ^{1}H–^{13}C HMBC NMR spectrum of 3 (400 MHz, toluene-d_8, 253 K).......... 12

Figure S6. Variable temperature ^{1}H and ^{31}P experiments of complex 3 recorded in 10 K increments in the stable temperature regime (193–293 K).......... 13

Figure S7. Variable temperature ^{1}H experiments of complex 3 recorded in 10 K increments in the stable temperature regime (193–293 K).......... 14

Figure S9. ^{13}C DEPTQ NMR spectrum of 4 (400 MHz, toluene-d_8, 278 K).......... 16

Figure S10. ^{31}P NMR spectrum of 4 (400 MHz, toluene-d_8, 278 K).......... 17

Figure S11. ^{1}H–^{13}C HSQC NMR spectrum of 4 (400 MHz, toluene-d_8, 278 K).......... 18

Figure S12. ^{1}H–^{13}C HMBC NMR spectrum of 4 (400 MHz, toluene-d_8, 278 K).......... 19

Figure S14. ^{13}C DEPTQ NMR spectrum of 5 (400 MHz, toluene-d_8, 278 K).......... 21

Figure S15. ^{31}P NMR spectrum of 5 (400 MHz, toluene-d_8, 278 K).......... 22

Figure S16. ^{1}H–^{13}C HSQC NMR spectrum of 5 (400 MHz, toluene-d_8, 278 K).......... 23

Figure S17. ^{1}H–^{13}C HMBC NMR spectrum of 5 (400 MHz, toluene-d_8, 278 K).......... 24

Figure S18. ^{1}H–^{31}P HSQC NMR spectrum of 5 (400 MHz, toluene-d_8, 278 K). 25

Figure S19. ^{31}P–^{31}P COSY NMR spectrum of 5 (400 MHz, toluene-d_8, 278 K). 26

Crystallographic Details 27

Table S1. Summary of Crystallographic Data for Compounds 3–5. 27

Figure S20. Structure of one of the two independent molecules of 3 in the solid state 28

Figure S21. Molecular structure of 4 in the solid state 29

Figure S22. Molecular structure of 5 in the solid state 30

Computational Details 31

General Considerations. 31

Figure S23. Calculated haptotropic rearrangement between 3 and 3', *via* 3'' over transition states TS_1 and TS_2. 32

Figure S24. Five most important ETS-NOCV deformation density contributions (isovalues = ±0.001–0.003 a.u.) to bonding between $((1)Ni)_2$ and P_4 in 3. 33

Figure S25. Frontier Kohn-Sham orbitals of 4 (isovalue = ±0.004 a.u.) in two different molecular orientations. 34

Figure S26. Four most important ETS-NOCV deformation density contributions (isovalues = ±0.001–0.003 a.u.) to bonding between $((1)Ni)_2$ and P_2 in 4. 35

References 36

Synthetic Details

General Considerations. Storage and manipulation of all compounds were performed under an argon atmosphere either in an MBRAUN glove box or using a double manifold high vacuum line. Toluene and pentane were dried and purified using an MBRAUN solvent purification system and stored in 500 mL thick-walled vessels over sodium/benzophenone ketyl. Benzene and tetrahydrofuran were dried over potassium, distilled, and stored over sodium/benzophenone ketyl in 500 mL thick walled vessels. All solvents were degassed and vacuum distilled prior to use. ^{1}H and ^{13}C NMR chemical shifts were referenced to residual solvent protons and naturally abundant ^{13}C resonances for all deuterated solvents: benzene-d_5 (7.16 ppm; ^{1}H) and benzene-d_6 (128.06 ppm; ^{13}C); toluene-d_7 (2.08, 6.97, 7.01, 7.09 ppm; ^{1}H) and toluene-d_8 (20.43, 125.13; 127.96; 128.87; 137.48 ppm; ^{13}C); tetrahydrofuran-d_7 (3.58 ppm; ^{1}H) and tetrahydrofuran-d_8 (67.21 ppm; ^{13}C).[1] Chemical shift assignments are based on ^{1}H, ^{13}C, ^{31}P, ^{1}H-^{13}C-HSQC, and ^{1}H-^{13}C-HMBC NMR experiments performed on Bruker 400 MHz spectrometers. Unless otherwise specified, the spectra were acquired at 298 K. X-ray crystallographic analyses were performed on a Nonius Kappa CCD diffractometer with samples coated in Paratone 8277 oil (Exxon) and mounted on a glass fiber loop. Complex **2** was prepared following literature methods. All other reagents were purchased from commercial suppliers and used as received. All elemental analyses were obtained by the Instrumentation Facility of the Department of Chemistry, University of Calgary.

Synthesis of {[(1)Ni]$_2$(μ_2:η^2,η^2-P_4)}, 3.[2] Nickel complex **2** (300 mg, 0.38 mmol) and half an equivalent of P_4 (23.7 mg, 0.19 mmol) were charged to a round bottom flask attached to a pressure-equalizing swivel frit. Pentane (50 mL) was condensed over this mixture and the reaction was stirred at 0 ºC for two hours. The micro-crystalline product was recovered by filtration and washed once with cold pentane (10 mL). The product was isolated as a light brown solid (204 mg, 0.28

mmol, 73%). The reaction was quickly processed, and the product was stored at low temperature to limit decomposition. **3** can also be prepared from reaction of **2** with an excess of P_4. Crystals suitable for diffraction experiments were grown from a concentrated pentane or THF solution at −40 ºC. Complex **3** had good solubility in benzene, toluene, diethyl ether, THF, and moderate solubility in *n*-pentane. ^{1}H NMR (400 MHz, toluene-d_8, 253 K) δ 0.26 (d, $^3J_{HH}$ = 6.9 Hz, 3H, CH(C**H**$_3$)$_2$), 0.38 (s, 3H, Si(C**H**$_3$)$_2$), 0.42 (s, 3H, Si(C**H**$_3$)$_2$), 0.50 (s, 3H, Si(C**H**$_3$)$_2$), 0.76 (d, $^3J_{HH}$ = 6.5 Hz, 3H, CH(C**H**$_3$)$_2$), 0.88 (d, $^3J_{HH}$ = 6.1 Hz, 3H, CH(C**H**$_3$)$_2$), 1.03 (s, 3H, Si(C**H**$_3$)$_2$), 1.06 (d, $^3J_{HH}$ = 6.5 Hz, 3H, CH(C**H**$_3$)$_2$), 1.15 (m, 9H, CH(C**H**$_3$)$_2$), 1.27 (d, $^3J_{HH}$ = 6.1 Hz, 3H, CH(C**H**$_3$)$_2$), 2.28 (sept, $^3J_{HH}$ = 6.9 Hz, 1H, C**H**(CH$_3$)$_2$), 2.87 (sept, $^3J_{HH}$ = 6.8 Hz, 1H, C**H**(CH$_3$)$_2$), 3.00 (m, $^3J_{HH}$ = 5.4 Hz, 2H, C**H**(CH$_3$)$_2$), 3.26 (d, $^3J_{HH}$ = 15.0 Hz, 1H, C**H**$_2$), 4.25 (d, $^3J_{HH}$ = 18.1 Hz, 1H, C**H**$_2$), 4.96 (d, $^3J_{HH}$ = 15.7 Hz, 1H, C**H**$_2$), 6.24 (d, $^3J_{HH}$ = 1.5 Hz, 1H, Im**H**), 6.40 (d, $^3J_{HH}$ = 1.5 Hz, 1H, Im**H**), 6.52 (d, $^3J_{HH}$ = 1.5 Hz, 1H, Im**H**), 6.79 (d, $^3J_{HH}$ = 1.6 Hz, 1H, Im**H**), 6.84 (m, 2H, Ar**H**), 7.06 (m, 1H, Ar**H**), 7.15–7.26 (m, 3H, Ar**H**), 8.15 (d, $^3J_{HH}$ = 17.1 Hz, 1H, C**H**$_2$). ^{13}C NMR (151 MHz, toluene-d_8, 253 K) δ −0.1 (s, Si(**C**H$_3$)$_2$), 0.7 (s, Si(**C**H$_3$)$_2$), 0.8 (s, Si(**C**H$_3$)$_2$), 1.1 (s, Si(**C**H$_3$)$_2$), 21.8 (s, CH(**C**H$_3$)$_2$), 22.5 (s, CH(**C**H$_3$)$_2$), 23.8 (s, CH(**C**H$_3$)$_2$), 24.2 (s, CH(**C**H$_3$)$_2$), 25.4 (s, CH(**C**H$_3$)$_2$), 26.0 (s, CH(**C**H$_3$)$_2$), 26.2 (s, CH(**C**H$_3$)$_2$), 27.9 (s, **C**H(CH$_3$)$_2$), 28.0 (s, **C**H(CH$_3$)$_2$), 28.5 (s, **C**H(CH$_3$)$_2$), 42.8 (s, **C**H$_2$), 52.5 (s, **C**H$_2$), 118.5 (s, **C**$_2$N$_2$C), 120.2 (s, **C**$_2$N$_2$C), 22.7 (s, *m/p*-**C**$_6$H$_3$), 123.1 (s, *m/p*-**C**$_6$H$_3$), 124.5 (s, **C**$_2$N$_2$C), 125.1 (s, **C**$_2$N$_2$C), 138.6 (s, *i*-**C**$_6$H$_3$), 139.8 (s, *i*-**C**$_6$H$_3$), 145.3 (s, *o*-**C**$_6$H$_3$), 145.6 (s, *o*-**C**$_6$H$_3$), 146.1 (s, *o*-**C**$_6$H$_3$), 148.1 (s, *o*-**C**$_6$H$_3$), 192.8 (s, N$_2$**C**), 202.7 (s, N$_2$**C**). ^{31}P NMR (162 MHz, toluene-d_8, 253 K) δ 44.9. Anal. calcd. for $C_{72}H_{108}N_8O_2Si_4Ni_2P_4(C_5H_{12})$: C 59.92; H 7.84; N 7.26. Found: C 59.84; H 7.73; N 7.17.

Synthesis of {[(1)Ni]$_2$(μ$_2$:η^2,η^2-P$_2$)}, 4. Nickel complex **2** (300 mg, 0.38 mmol) and a quarter equivalent of P_4 (11.9 mg, 0.095 mmol) were charged to a round bottom flask attached to a

pressure-equalizing swivel frit. Pentane (50 mL) was condensed over this mixture and the reaction was stirred at 0 ºC for two hours. The reaction mixture was then filtered, the solvents removed in vacuo, and the residue was triturated 6 times with pentane (6 × 10 mL). The residue was extracted into pentane (20 mL) and crystallized at −40 ºC. The product was purified by concentrating and crystallizing the mother liquor once more yielding orange-red crystals (164 mg, 0.23 mmol, 61 %). **4** can also be prepared from reaction of **2** with an excess of P_4 in toluene or by reaction of two equivalents of **2** with complex **3** in toluene. Complex **4** was prepared reproducibly and shows high solubility in most organic solvents, including *n*-pentane. Single-crystals were obtained by diffusing pentane into a toluene solution of this complex at −40 °C. ^{1}H NMR (400 MHz, toluene-d_8, 278 K) δ 0.16 (s, 3H, Si(C**H**$_3$)$_2$), 0.19 (s, 3H, Si(C**H**$_3$)$_2$), 0.22 (s, 3H, Si(C**H**$_3$)$_2$), 0.41 (s, 3H, Si(C**H**$_3$)$_2$), 0.73 (d, $^{3}J_{HH}$ = 7.1 Hz, 3H, CH(C**H**$_3$)$_2$), 0.79 (d, $^{3}J_{HH}$ = 6.8 Hz, 3H, CH(C**H**$_3$)$_2$), 0.81 (d, $^{3}J_{HH}$ = 7.4 Hz, 3H, CH(C**H**$_3$)$_2$), 1.01 (d, $^{3}J_{HH}$ = 6.8 Hz, 3H, CH(C**H**$_3$)$_2$), 1.09 (d, $^{3}J_{HH}$ = 6.1 Hz, 1H, CH(C**H**$_3$)$_2$), 1.22 (d, $^{3}J_{HH}$ = 6.3 Hz, 3H, CH(C**H**$_3$)$_2$), 1.30 (d, $^{3}J_{HH}$ = 6.1 Hz, 6H, CH(C**H**$_3$)$_2$), 2.54 (sept, $^{3}J_{HH}$ = 7.3 Hz, 1H, C**H**(CH$_3$)$_2$), 2.75 (sept, $^{3}J_{HH}$ = 6.2 Hz, 1H, C**H**(CH$_3$)$_2$), 2.88 (sept, $^{3}J_{HH}$ = 6.8 Hz, 1H, C**H**(CH$_3$)$_2$), 3.00 (d, $^{3}J_{HH}$ = 15.1 Hz, 1H, C**H**$_2$), 3.09 (d, $^{3}J_{HH}$ = 16.6 Hz, 1H, C**H**$_2$), 3.73 (sept, $^{3}J_{HH}$ = 6.2 Hz, 1H, C**H**(CH$_3$)$_2$), 5.01 (d, $^{3}J_{HH}$ = 14.6 Hz, 1H, C**H**$_2$), 5.47 (d, $^{3}J_{HH}$ = 16.1 Hz, 1H, C**H**$_2$), 6.05 (d, $^{3}J_{HH}$ = 1.2 Hz, 1H, Im**H**), 6.14 (d, $^{3}J_{HH}$ = 1.5 Hz, 1H, Im**H**), 6.32 (d, $^{3}J_{HH}$ = 1.6 Hz, 1H, Im**H**), 6.48 (d, $^{3}J_{HH}$ = 1.6 Hz, 1H, Im**H**), 6.94 (dd, $^{3}J_{HH}$ = 7.8 Hz, $^{4}J_{HH}$ = 1.2 Hz, 1H, *m*-C$_6$**H**$_3$), 7.11 (m, 1H, *m/p*-C$_6$**H**$_3$), 7.18 (m, 2H, *m/p*-C$_6$**H**$_3$), 7.31 (dd, $^{3}J_{HH}$ = 7.8 Hz, $^{4}J_{HH}$ = 1.2 Hz, 1H, *m*-C$_6$**H**$_3$), 7.38 (t, $^{3}J_{HH}$ = 7.7 Hz, 1H, *p*-C$_6$**H**$_3$). ^{13}C NMR (151 MHz, toluene-d_8, 278 K) δ −1.2 (s, Si(CH$_3$)$_2$), 1.0 (s, Si(**C**H$_3$)$_2$), 1.1 (s, Si(**C**H$_3$)$_2$), 2.0 (s, Si(**C**H$_3$)$_2$), 23.1 (s, CH(**C**H$_3$)$_2$), 23.3 (s, CH(**C**H$_3$)$_2$), 23.9 (s, CH(**C**H$_3$)$_2$), 24.9 (s, CH(**C**H$_3$)$_2$), 25.4 (s, CH(**C**H$_3$)$_2$), 25.6 (s, CH(**C**H$_3$)$_2$), 26.0 (s, CH(**C**H$_3$)$_2$), 27.5 (s, **C**H(CH$_3$)$_2$), 27.7 (s, **C**H(CH$_3$)$_2$), 28.0 (s, **C**H(CH$_3$)$_2$), 28.4 (s,

$\mathbf{C}H(CH_3)_2$), 43.1 (s, $\mathbf{C}H_2$), 44.2 (s, $\mathbf{C}H_2$), 118.4 (s, $\mathbf{C}_2N_2C$), 119.8 (s, $\mathbf{C}_2N_2C$), 122.6 (s, *m*-$\mathbf{C}_6H_3$), 123.1 (s, *m/p*-$\mathbf{C}_6H_3$), 123.5 (s, *m/p*-$\mathbf{C}_6H_3$), 124.3 (s, $\mathbf{C}_2N_2C$), 124.5 (s, $\mathbf{C}_2N_2C$), 125.5 (s, *m*-$\mathbf{C}_6H_3$), 128.6 (s, *m/p*-$\mathbf{C}_6H_3$), 128.7 (s, *p*-$\mathbf{C}_6H_3$), 139.4 (s, *i*-$\mathbf{C}_6H_3$), 140.0 (s, *i*-$\mathbf{C}_6H_3$), 145.1 (s, *o*-$\mathbf{C}_6H_3$) , 145.4 (s, *o*-$\mathbf{C}_6H_3$), 146.9 (s, *o*-$\mathbf{C}_6H_3$), 149.2 (s, *o*-$\mathbf{C}_6H_3$), 198.2 (s, $N_2\mathbf{C}$), 208.1 (s, $N_2\mathbf{C}$). ^{31}P NMR (162 MHz, toluene-d_8, 278 K) δ 144.7. Anal. calcd. for $C_{72}H_{108}N_8O_2Si_4Ni_2P_2$: C 61.36; H 7.72; N 7.95. Found: C 60.02; H 8.01; N 7.17.

Synthesis of {[(1)Ni]$P_7(SiMe_3)_3$}, 5. To a stirred suspension of **2** (300 mg, 0.38 mmol) in pentane (10 mL) a solution of tris(trimethylsilyl)heptaphosphine (167 mg, 0.38 mmol) in pentane (5 mL) was added dropwise, at room temperature. The reaction was allowed to stir for 15 min and the suspension was filtered. The product was crystallized at −40 ºC from the filtrate, yielding **5** as a bronze crystalline solid (313 mg, 0.28 mmol, 74 %) with high solubility in most organic solvents, including *n*-pentane. ^{1}H NMR (400 MHz, toluene-d_8, 278 K): δ 0.10 (s, 3H, $Si(C\mathbf{H}_3)_2$), 0.11 (s, 3H, $Si(C\mathbf{H}_3)_2$), 0.23 (d, $^3J_{HP}$ = 3.7 Hz, 9H, $P(Si(C\mathbf{H}_3)_3)$), 0.26 (d, $^3J_{HP}$ = 4.2 Hz, 9H, $P(Si(C\mathbf{H}_3)_3)$), 0.35 (s, 3H, $Si(C\mathbf{H}_3)_2$), 0.44 (d, $^3J_{HP}$ = 4.3 Hz, 9H, $P(Si(C\mathbf{H}_3)_3)$), 0.51 (s, 3H, $Si(C\mathbf{H}_3)_2$), 0.70 (d, $^3J_{HH}$ = 7.2 Hz, 6H, $CH(C\mathbf{H}_3)_2$), 0.89 (d, $^3J_{HH}$ = 6.9 Hz, 3H, $CH(C\mathbf{H}_3)_2$), 1.09 (d, $^3J_{HH}$ = 6.4 Hz, 3H, $CH(C\mathbf{H}_3)_2$), 1.13 (d, $^3J_{HH}$ = 6.7 Hz, 3H, $CH(C\mathbf{H}_3)_2$), 1.39 (d, $^3J_{HH}$ = 6.2 Hz, 3H, $CH(C\mathbf{H}_3)_2$), 1.42 (d, $^3J_{HH}$ = 6.7 Hz, 3H, $CH(C\mathbf{H}_3)_2$), 1.57 (d, $^3J_{HH}$ = 6.7 Hz, 3H, $CH(C\mathbf{H}_3)_2$), 2.23 (sept, $^3J_{HH}$ = 6.3 Hz, 1H, $C\mathbf{H}(CH_3)_2$), 2.37 (sept, $^3J_{HH}$ = 6.3 Hz, 1H, $C\mathbf{H}(CH_3)_2$), 2.81 (d, $^2J_{HH}$ = 16.3 Hz, 1H, $C\mathbf{H}_2$), 3.01 (sept, $^3J_{HH}$ = 6.6 Hz, 1H, $C\mathbf{H}(CH_3)_2$), 3.51 (d, $^2J_{HH}$ = 14.7 Hz, 1H, $C\mathbf{H}_2$), 4.36 (sept, $^3J_{HH}$ = 6.6 Hz, 1H, $C\mathbf{H}(CH_3)_2$), 4.44 (d, $^2J_{HH}$ = 16.9 Hz, 1H, $C\mathbf{H}_2$), 5.93 (d, $^3J_{HH}$ = 1.6 Hz, 1H, Im**H**), 6.29 (d, $^3J_{HH}$ = 1.7 Hz, 1H, Im**H**), 6.47 (d, $^3J_{HH}$ = 1.6 Hz, 1H, Im**H**), 6.58 (d, $^3J_{HH}$ = 1.7 Hz, 1H, Im**H**), 6.98 (m, 1H, *m,p*-$C_6\mathbf{H}_3$), 7.01 (m, 1H, *m,p*-$C_6\mathbf{H}_3$), 7.27 (m, 2H, *m,p*-$C_6\mathbf{H}_3$), 7.45 (t, $^3J_{HH}$ = 7.8 Hz, 1H, *p*-$C_6\mathbf{H}_3$), 7.57 (dd, $^3J_{HH}$ = 7.8 Hz, $^4J_{HH}$ = 1.1 Hz, 1H, *m*-$C_6\mathbf{H}_3$), 7.63 (dd, $^2J_{HH}$ = 16.7 Hz, $^{TS}J_{PH}$

= 5.3 Hz, 1H, C**H**$_2$). ^{13}C NMR (151 MHz, toluene-*d*$_8$, 278 K) δ 0.6 (s, br, Si(**C**H$_3$)$_2$), 0.7 (s, Si(**C**H$_3$)$_2$), 0.8 (d, $^{TS}J_{CP}$ = 3.5 Hz, Si(**C**H$_3$)$_2$), 0.9 (s, Si(**C**H$_3$)$_2$), 1.2 (d,br, $^2J_{CP}$ = 11.1 Hz, Si(**C**H$_3$)$_3$), 3.1 (d,br, $^2J_{CP}$ = 9.8 Hz, Si(**C**H$_3$)$_3$), 3.6 (d,br, $^2J_{CP}$ = 11.9 Hz, Si(**C**H$_3$)$_3$), 21.9 (s, CH(**C**H$_3$)$_2$), 24.7 (m, CH(**C**H$_3$)$_2$), 25.9 (s, CH(**C**H$_3$)$_2$), 26.6 (s, CH(**C**H$_3$)$_2$), 27.1 (s, CH(**C**H$_3$)$_2$), 27.6 (s, **C**H(CH$_3$)$_2$), 27.8 (s, CH(**C**H$_3$)$_2$), 28.4 (s, **C**H(CH$_3$)$_2$), 28.5 (s, **C**H(CH$_3$)$_2$), 29.3 (s, **C**H(CH$_3$)$_2$), 42.7 (s, **C**H$_2$), 48.3 (dd, $^{TS}J_{CP}$ = 20.9 Hz, $^{TS}J_{CP}$ = 9.2 Hz, **C**H$_2$), 117.8 (s, **C**$_2$N$_2$C), 120.9 (s, **C**$_2$N$_2$C), 123.4 (s, *m,p*-**C**$_6$H$_3$), 124.8 (s, *m*-**C**$_6$H$_3$), 125.7 (s, *m,p*-**C**$_6$H$_3$), 125.9 (s, **C**$_2$N$_2$C), 126.5 (s, **C**$_2$N$_2$C), 128.9 (s, *m,p*-**C**$_6$H$_3$), 129.1 (s, *p*-**C**$_6$H$_3$), 129.4 (s, *m,p*-**C**$_6$H$_3$), 137.7 (s, *i*-**C**$_6$H$_3$), 138.1 (s, *i*-**C**$_6$H$_3$), 144.9 (s, *o*-**C**$_6$H$_3$), 145.9 (s, *o*-**C**$_6$H$_3$), 147.2 (s, *o*-**C**$_6$H$_3$), 147.5 (s, *o*-**C**$_6$H$_3$), 190.8 (d, $^2J_{CP}$ = 20.2 Hz, N$_2$**C**), 198.0 (d, $^2J_{CP}$ = 8.4 Hz, N$_2$**C**). ^{1}P NMR (162 MHz, toluene-*d*$_8$, 278 K) δ −119.1 (m, P1), −68.49 (m, P3), −55.5 (m, P5), −32.2 (m, P4), −31.6 (m, P7), −16.21 (m, P2), 18.4 (m, P6). Anal. calcd. for $C_{45}H_{81}N_4OSi_5NiP_7$: C 48.69; H 7.35; N 5.05. Found: C 48.64; H 7.30; N 4.57

The most notable resonance in the ^{1}H NMR spectrum of **5** corresponds to the methylene proton H4a on the ligand backbone (Figure S22), which is involved in the anagostic interaction with the metal. This resulted in a dramatic downfield shift to 7.63 ppm (*vs.* 4.44 ppm for the geminal counterpart H4b). The close proximity of C4 to P3 and P5 (3.694(a) and 3.456(6) Å, respectively, Figure 4) leads to through-space ^{31}P–^{13}C coupling (9 and 21 Hz),[3] as well as ^{31}P–^{1}H coupling (5 Hz for P3–H4a), as confirmed by the ^{1}H–^{31}P HSQC spectrum (Figure S18).

NMR spectra

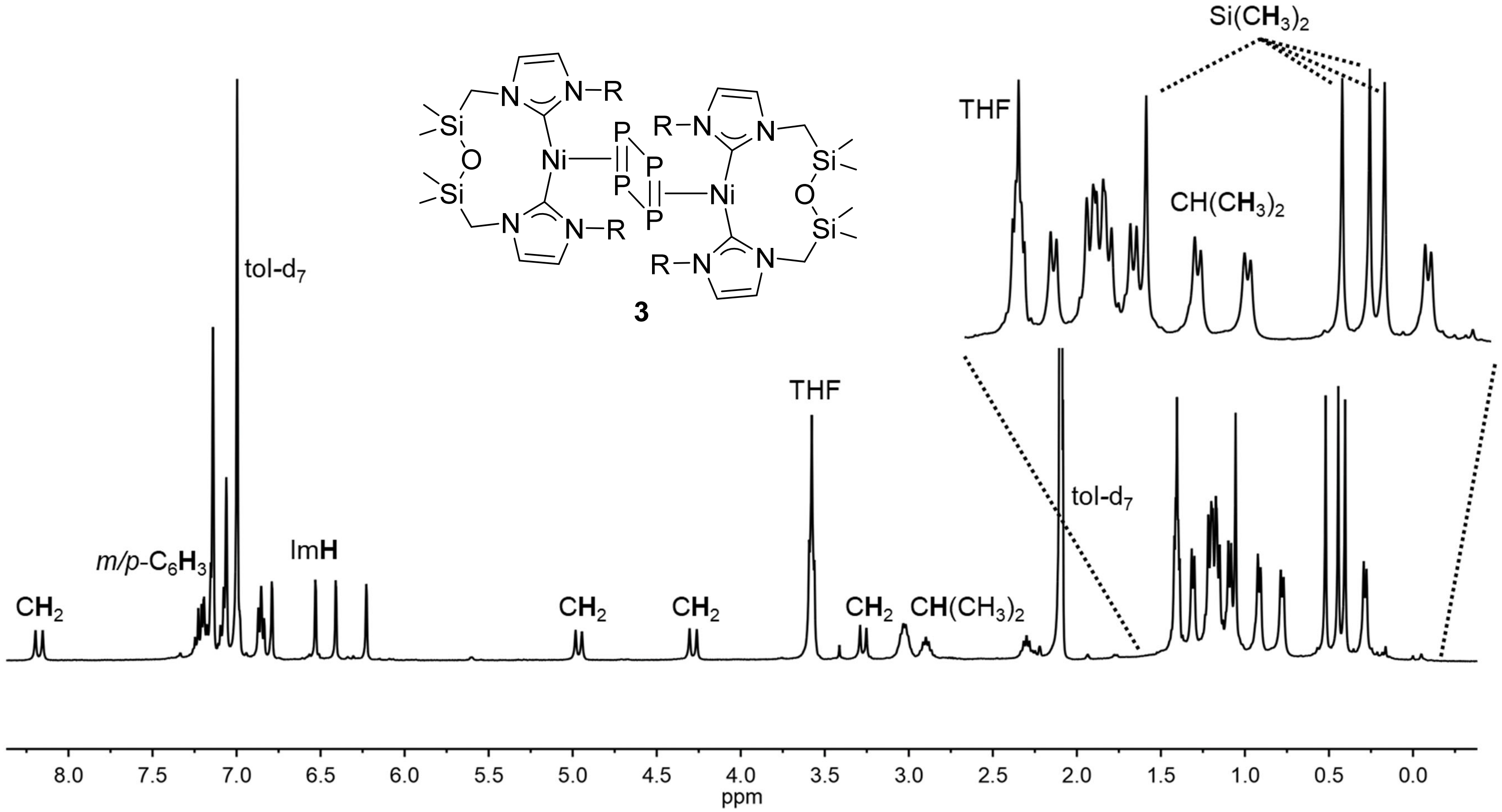

Figure S1. ^{1}H NMR spectrum of **3** (400 MHz, toluene-d_8, 253 K).

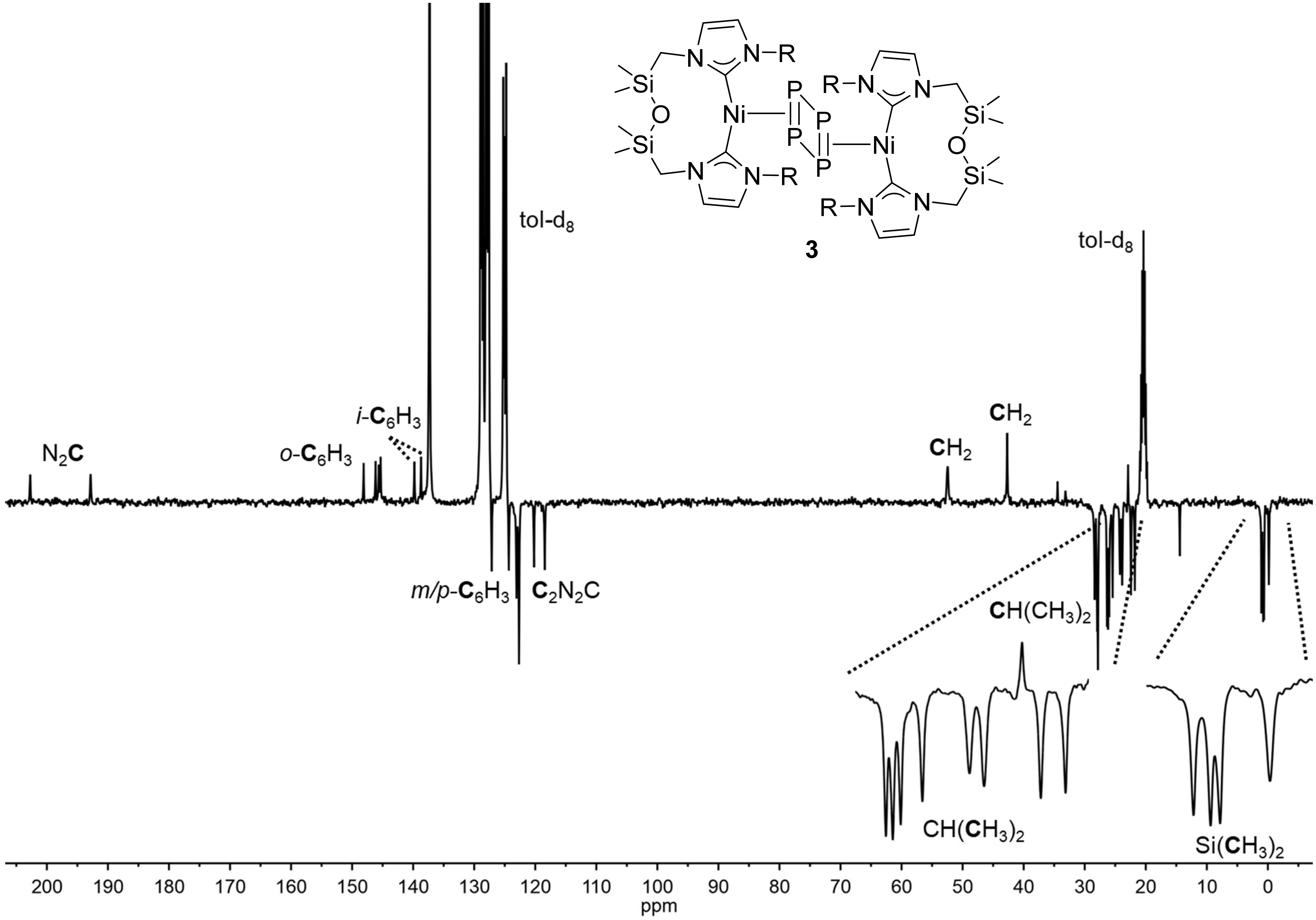

Figure S2. ^{13}C DEPTQ NMR spectrum of **3** (400 MHz, toluene-*d*$_8$, 253 K).

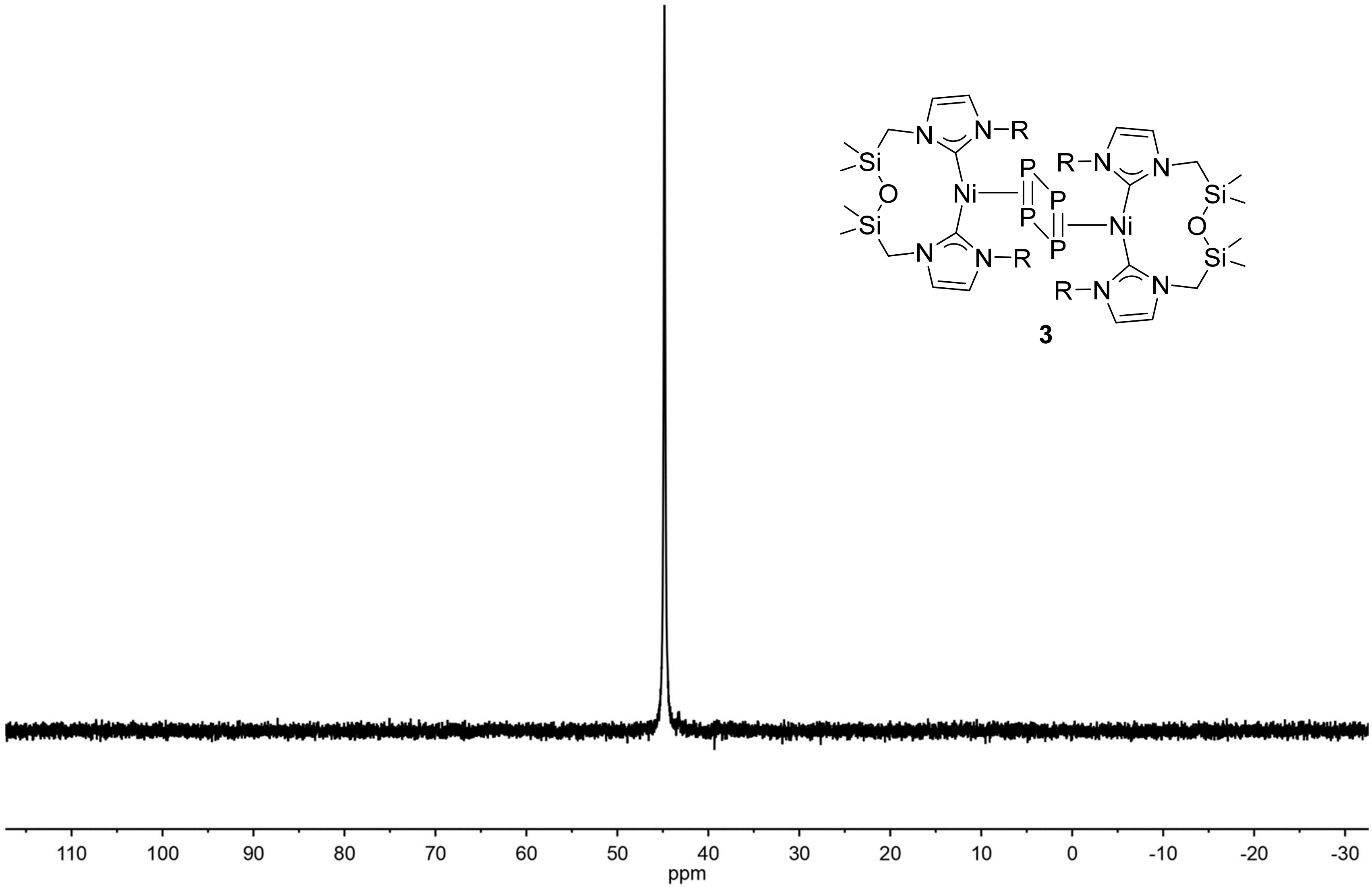

Figure S3. ^{31}P NMR spectrum of **3** (400 MHz, toluene-d_8, 293 K).

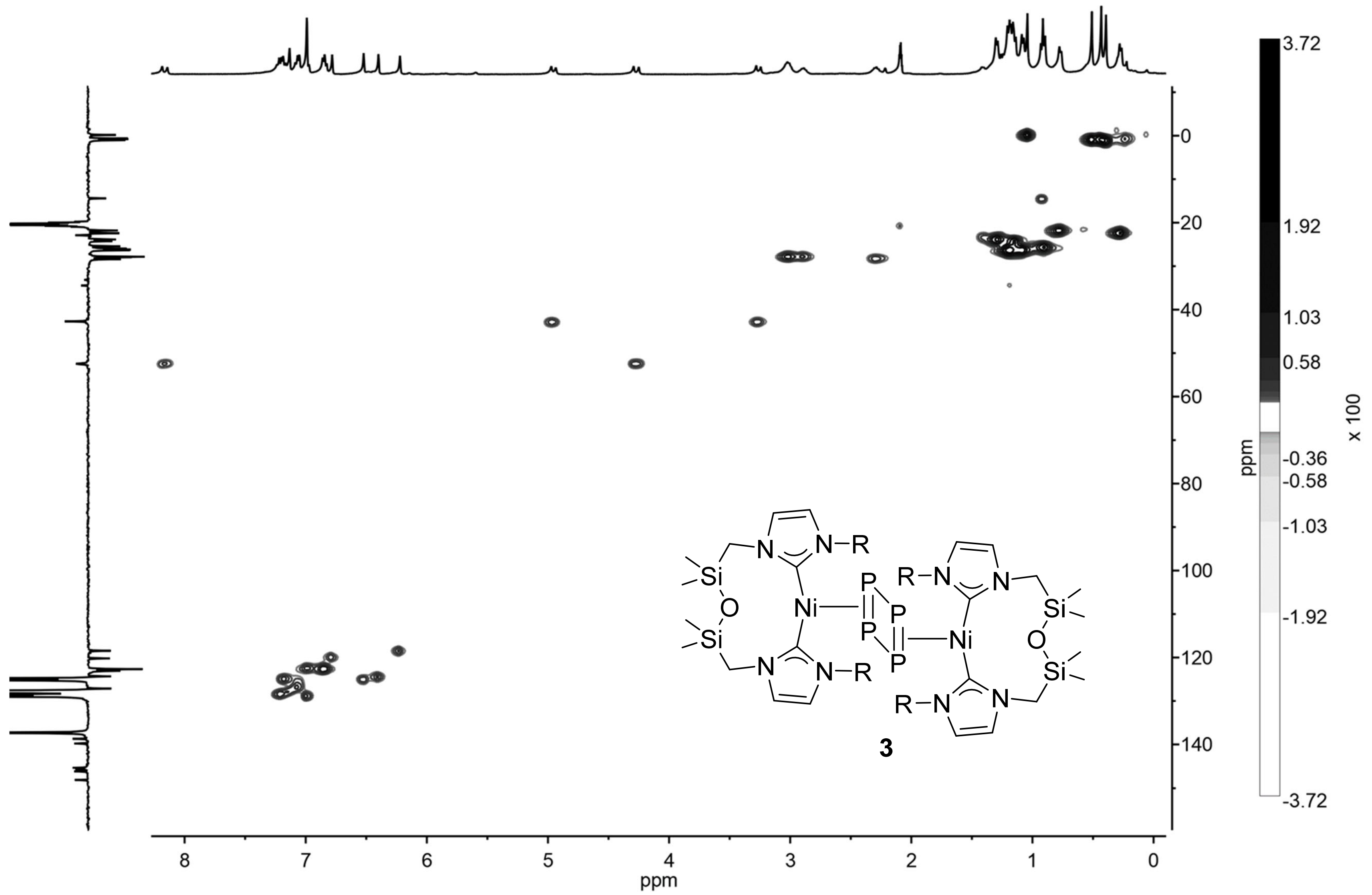

Figure S4. ^1H–^{13}C HSQC NMR spectrum of **3** (400 MHz, toluene-d_8, 253 K).

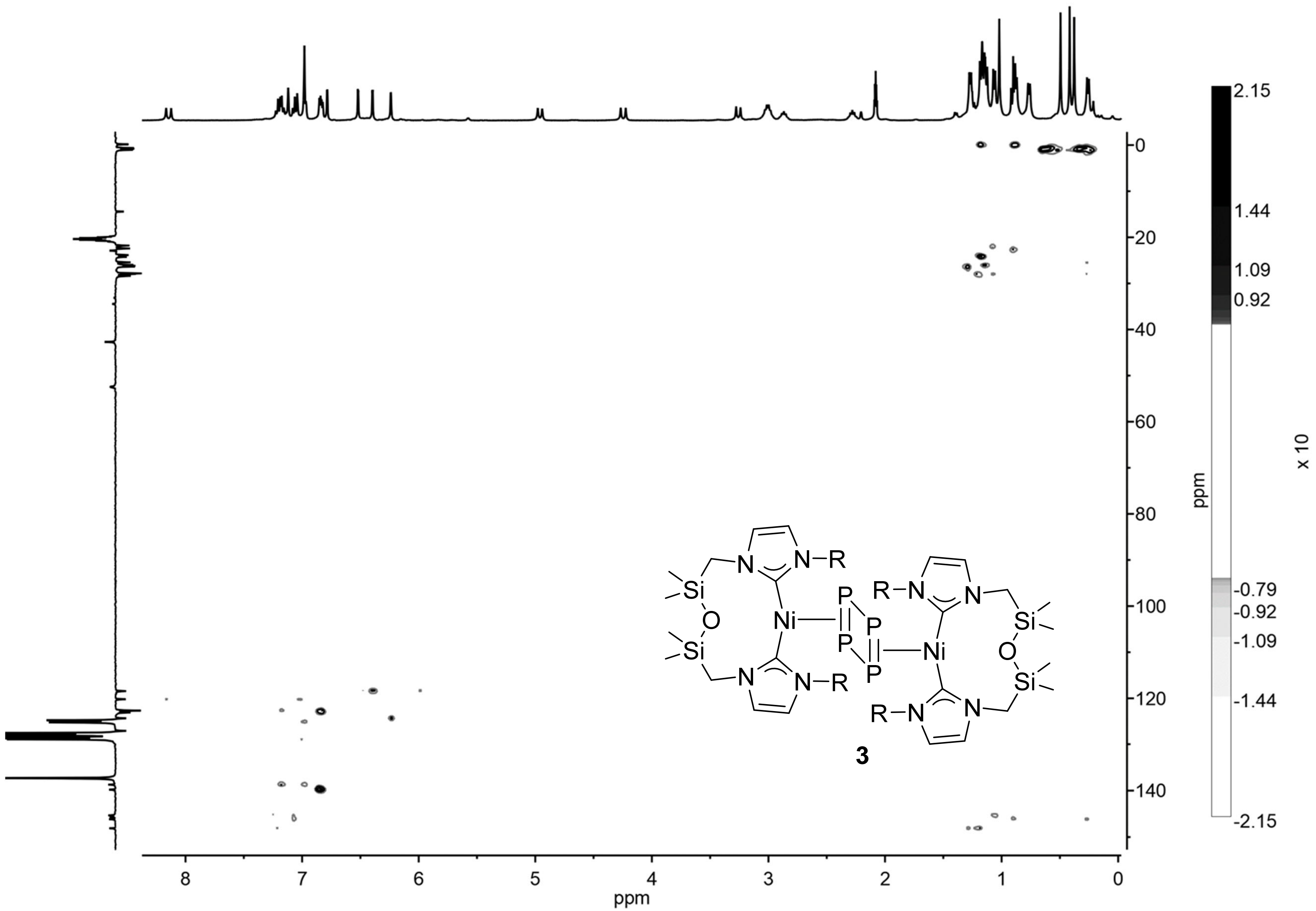

Figure S5. ^{1}H–^{13}C HMBC NMR spectrum of **3** (400 MHz, toluene-*d*8, 253 K).

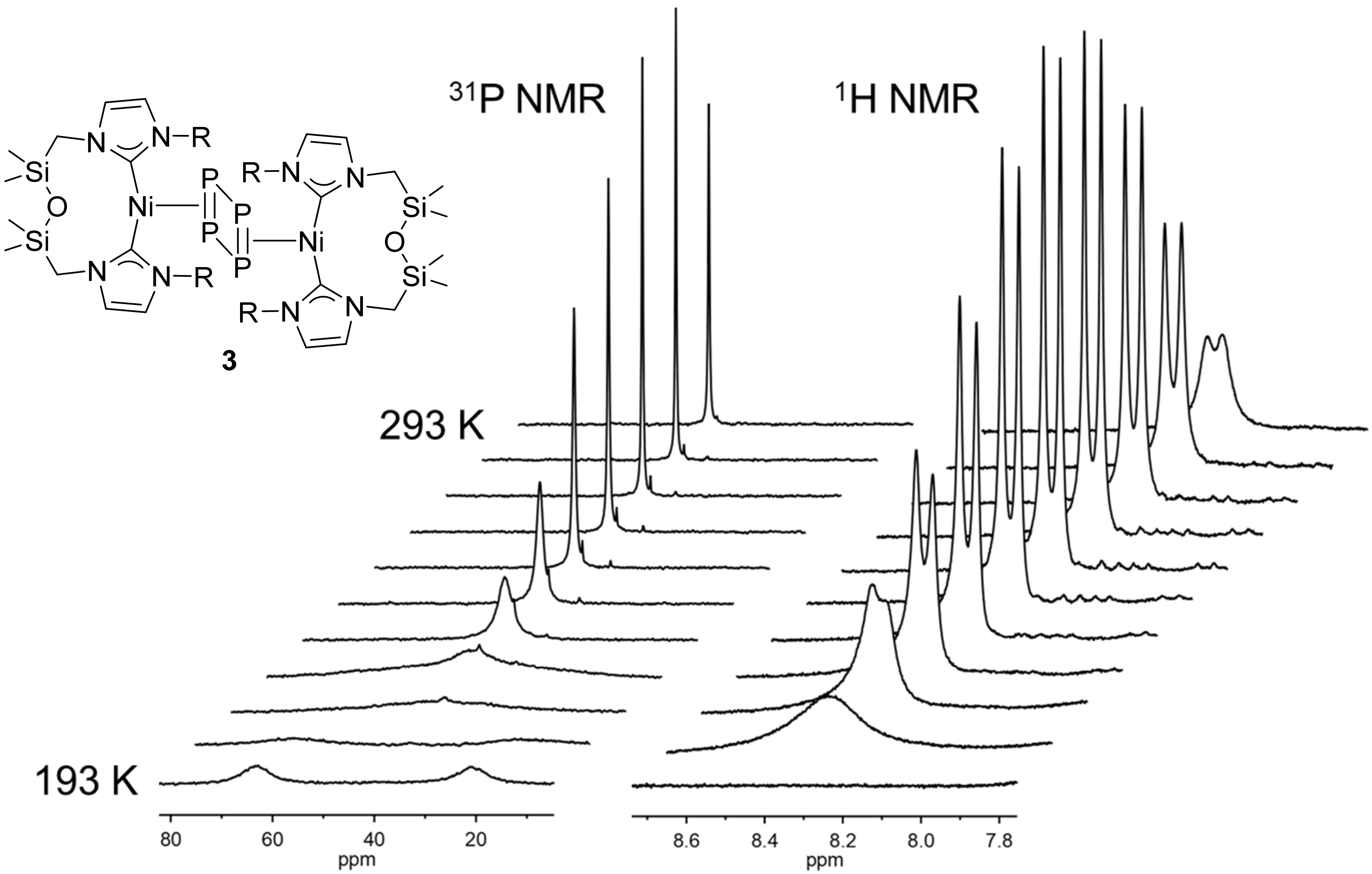

Figure S6. Variable temperature ^{1}H and ^{31}P experiments of complex **3** recorded in 10 K increments in the stable temperature regime (193–293 K). The ^{1}H NMR spectrum shown below characterizes the anagostically defined methylene fragment

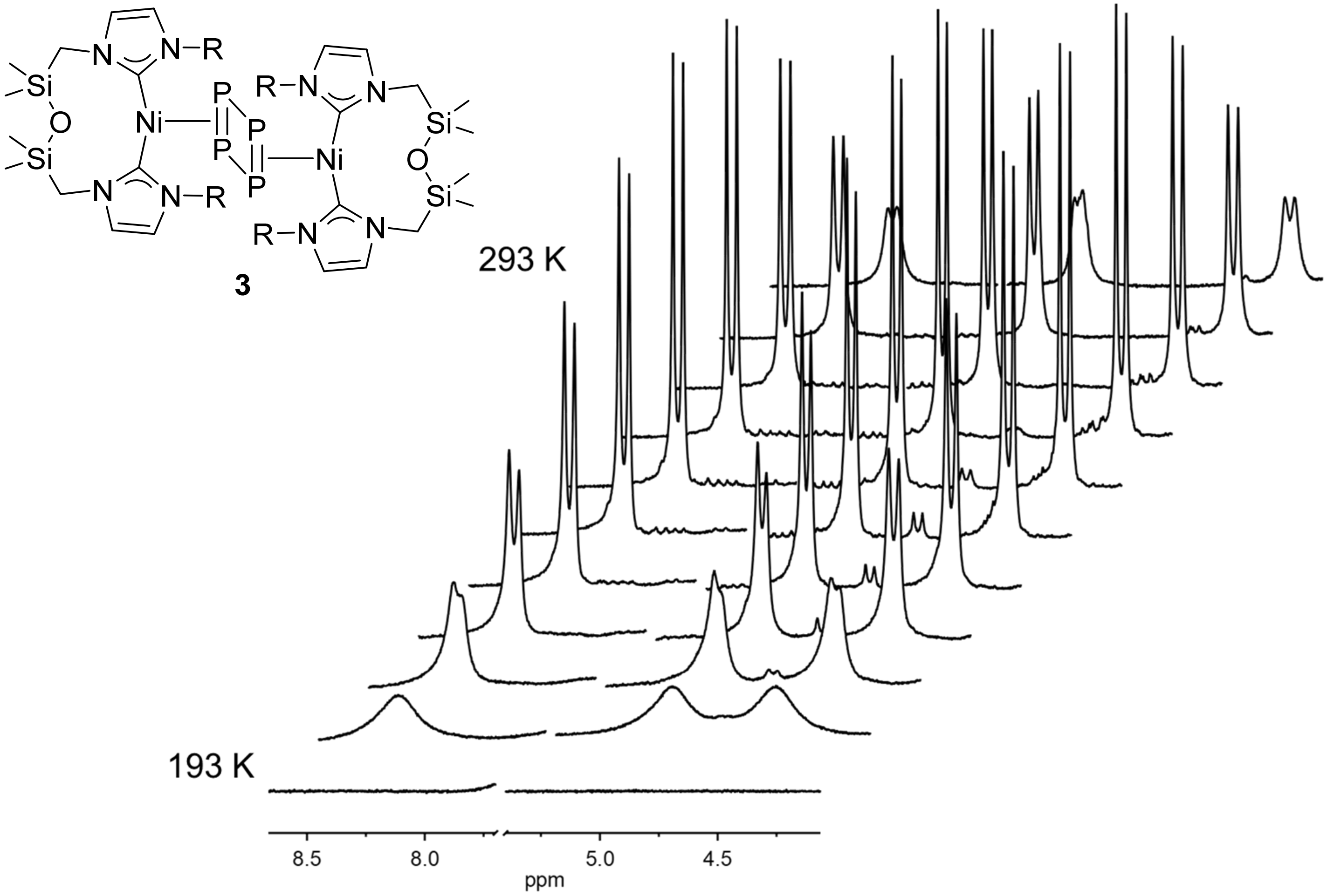

Figure S7. Variable temperature ^{1}H experiments of complex **3** recorded in 10 K increments in the stable temperature regime (193–293 K). The best resolved methylene resonances are shown below.

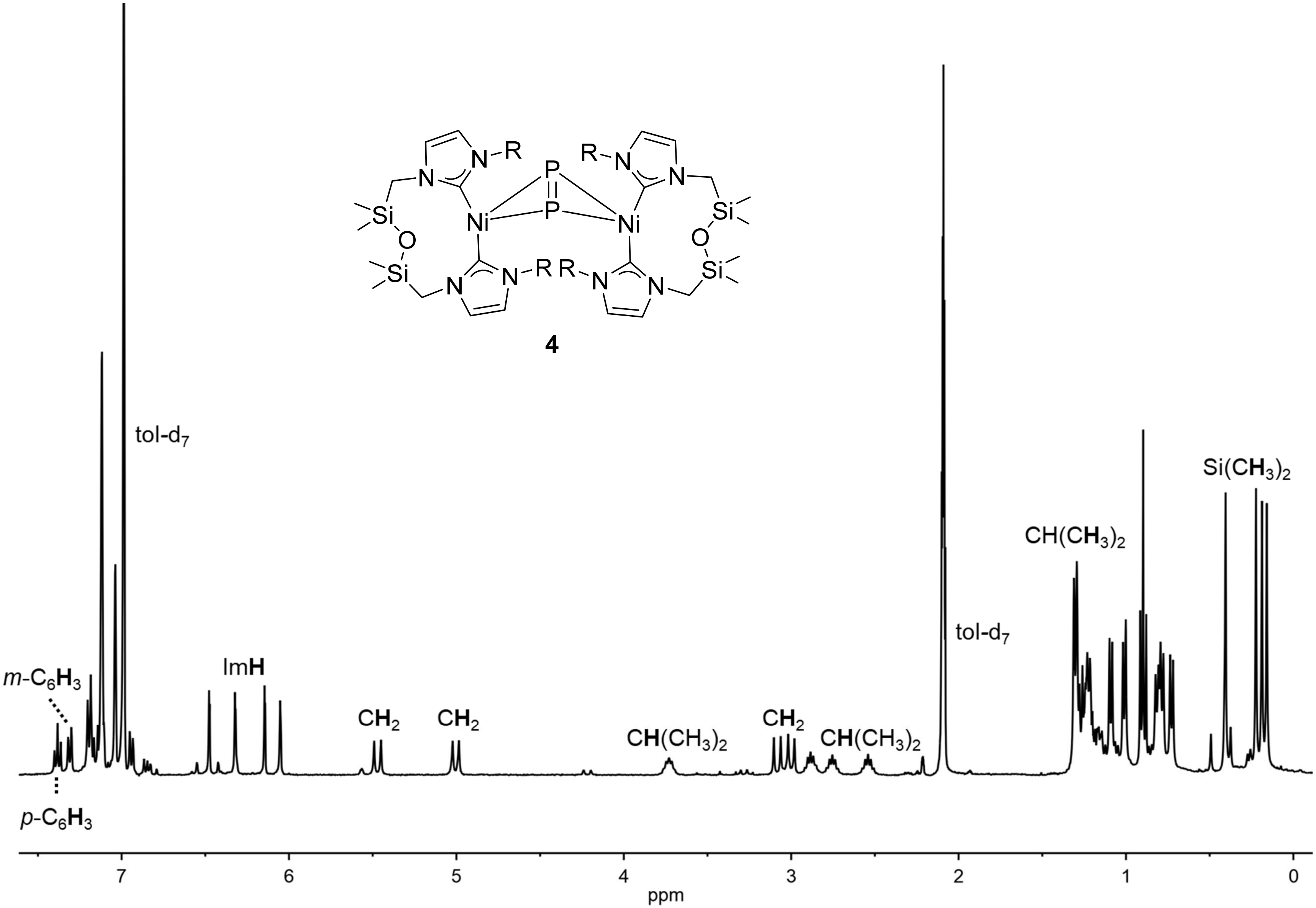

Figure S8. ^{1}H NMR spectrum of **4** (400 MHz, toluene-d_8, 278 K).

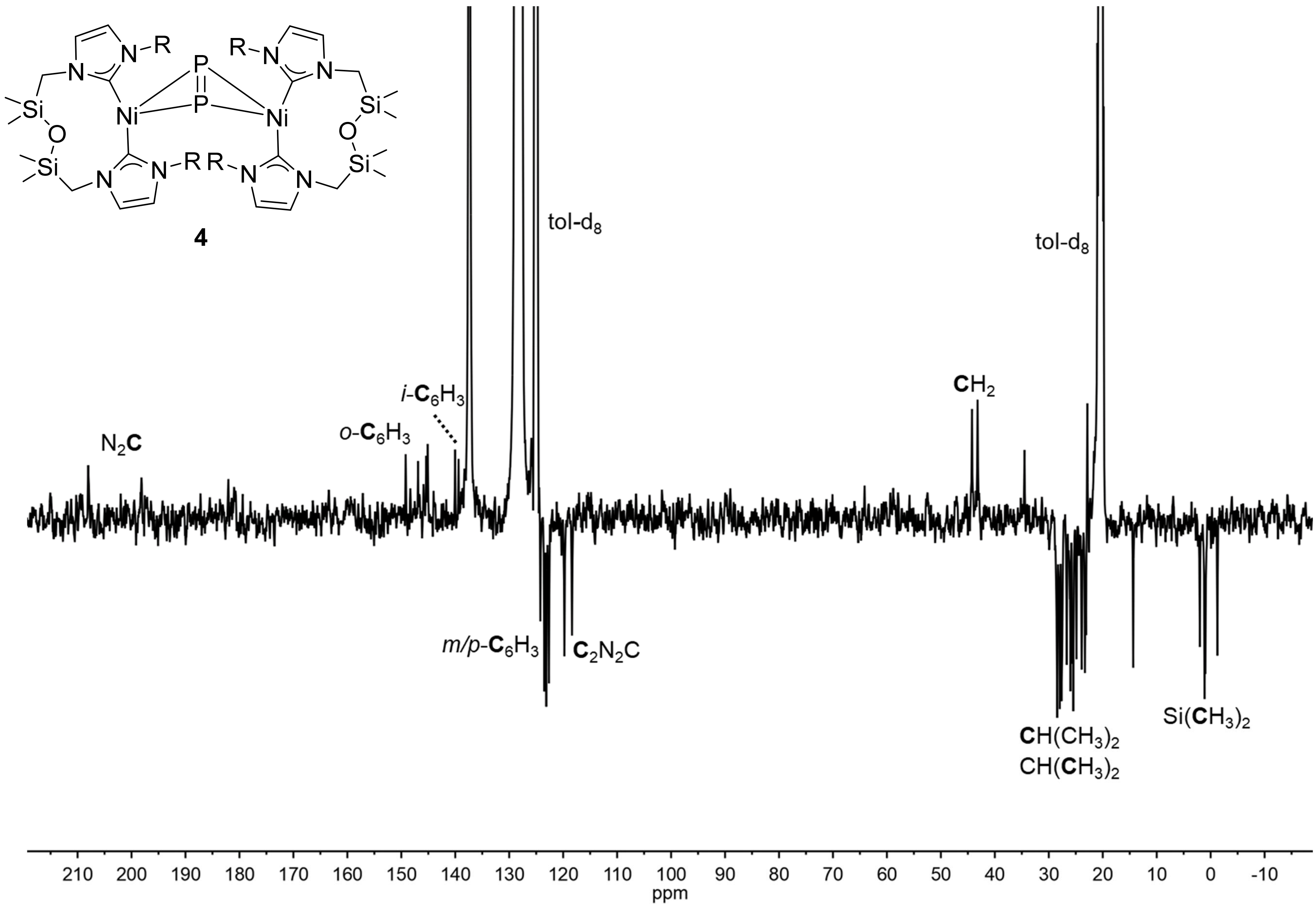

Figure S9. ^{13}C DEPTQ NMR spectrum of **4** (400 MHz, toluene-d_8, 278 K).

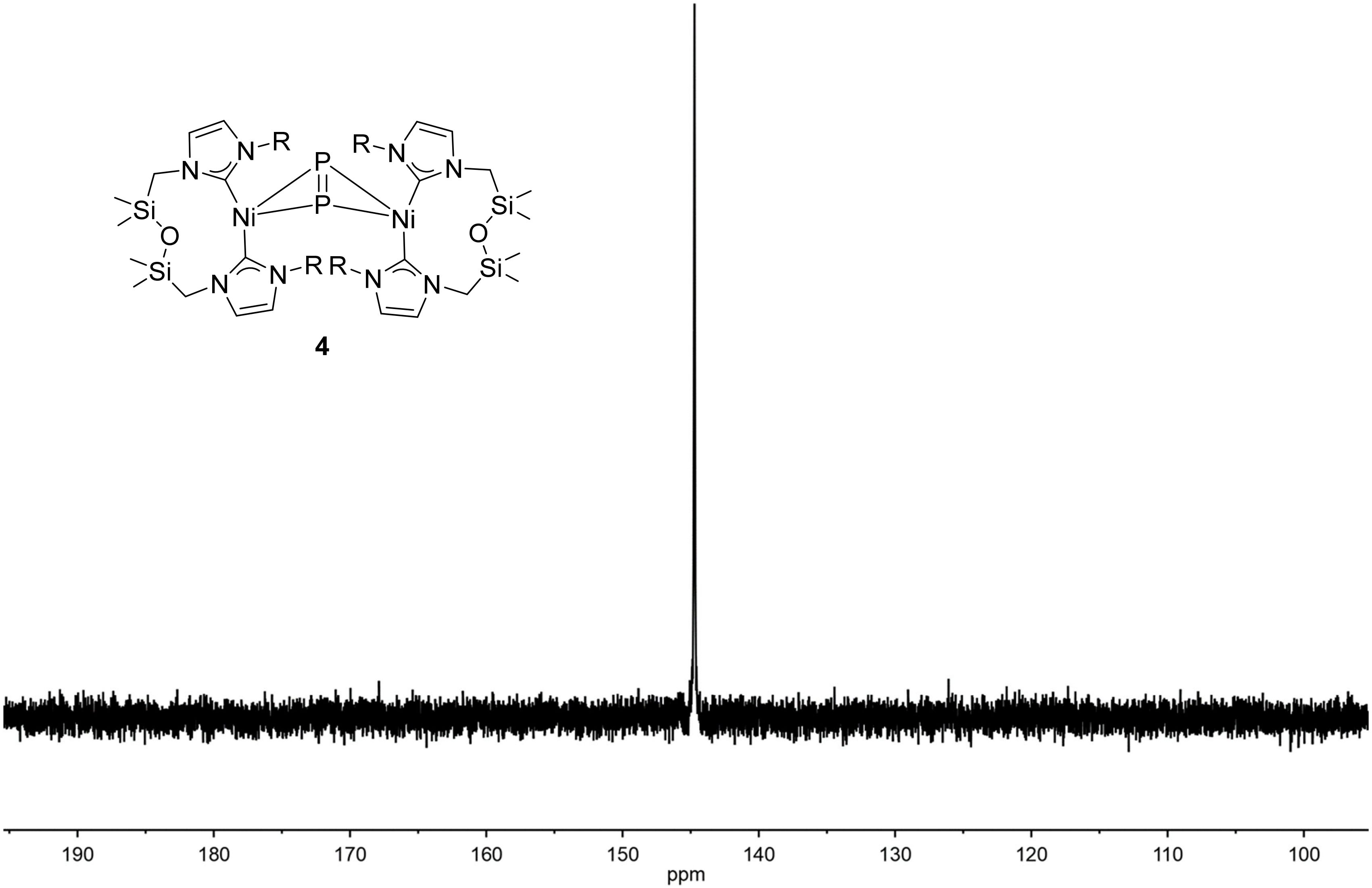

Figure S10. ^{31}P NMR spectrum of **4** (400 MHz, toluene-d_8, 278 K).

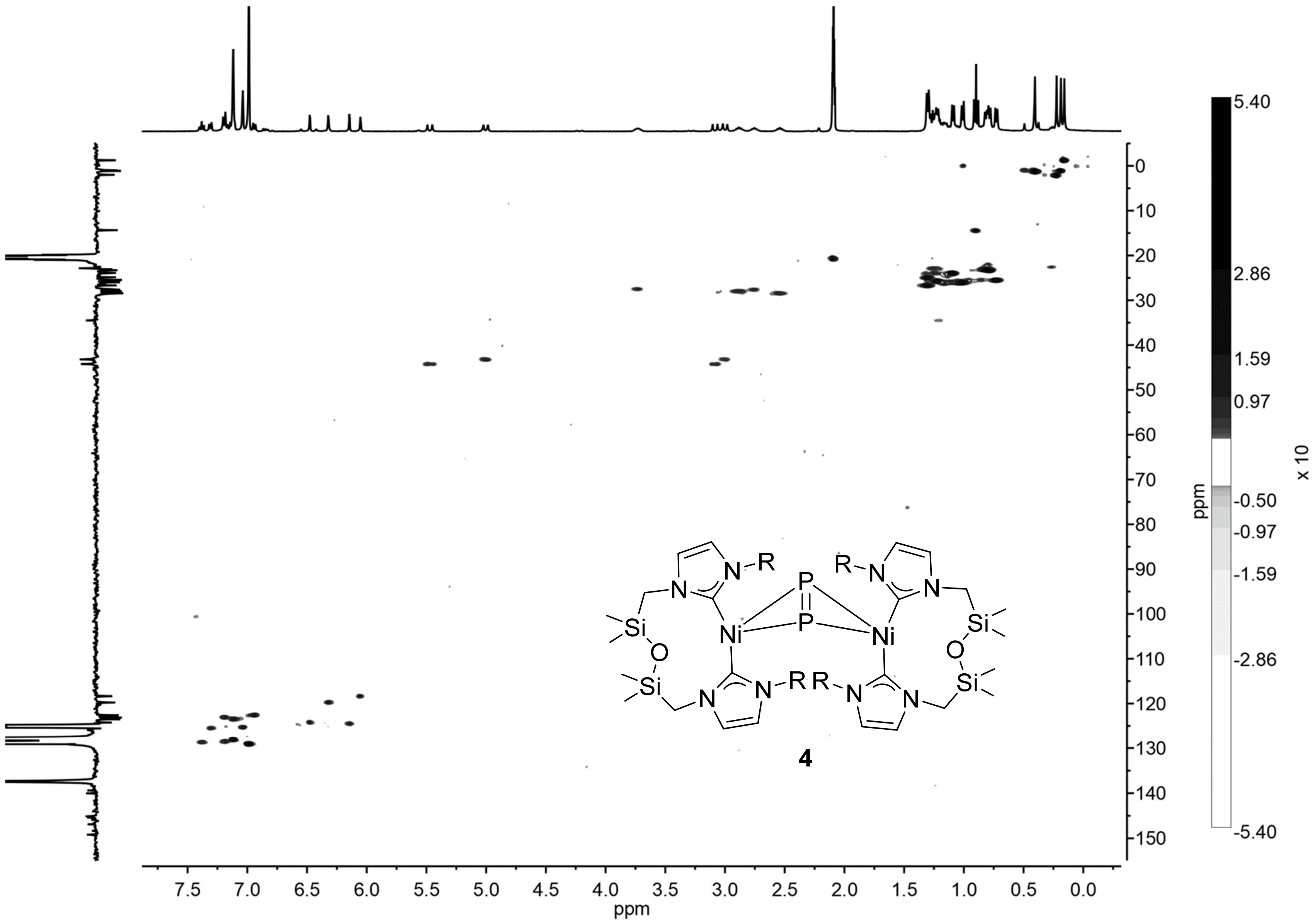

Figure S11. ^1H–^{13}C HSQC NMR spectrum of **4** (400 MHz, toluene-d_8, 278 K).

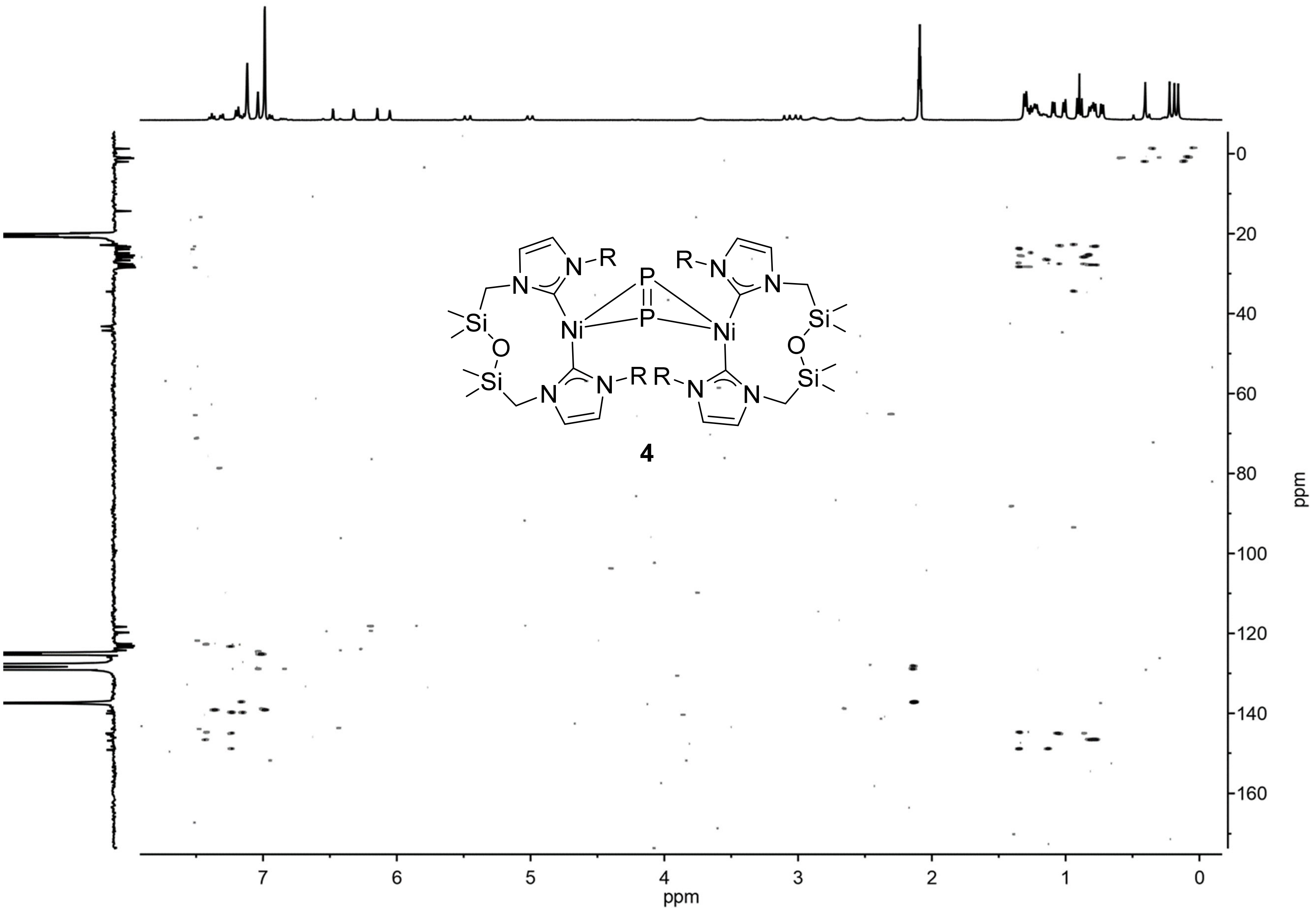

Figure S12. ^{1}H–^{13}C HMBC NMR spectrum of **4** (400 MHz, toluene-d_8, 278 K).

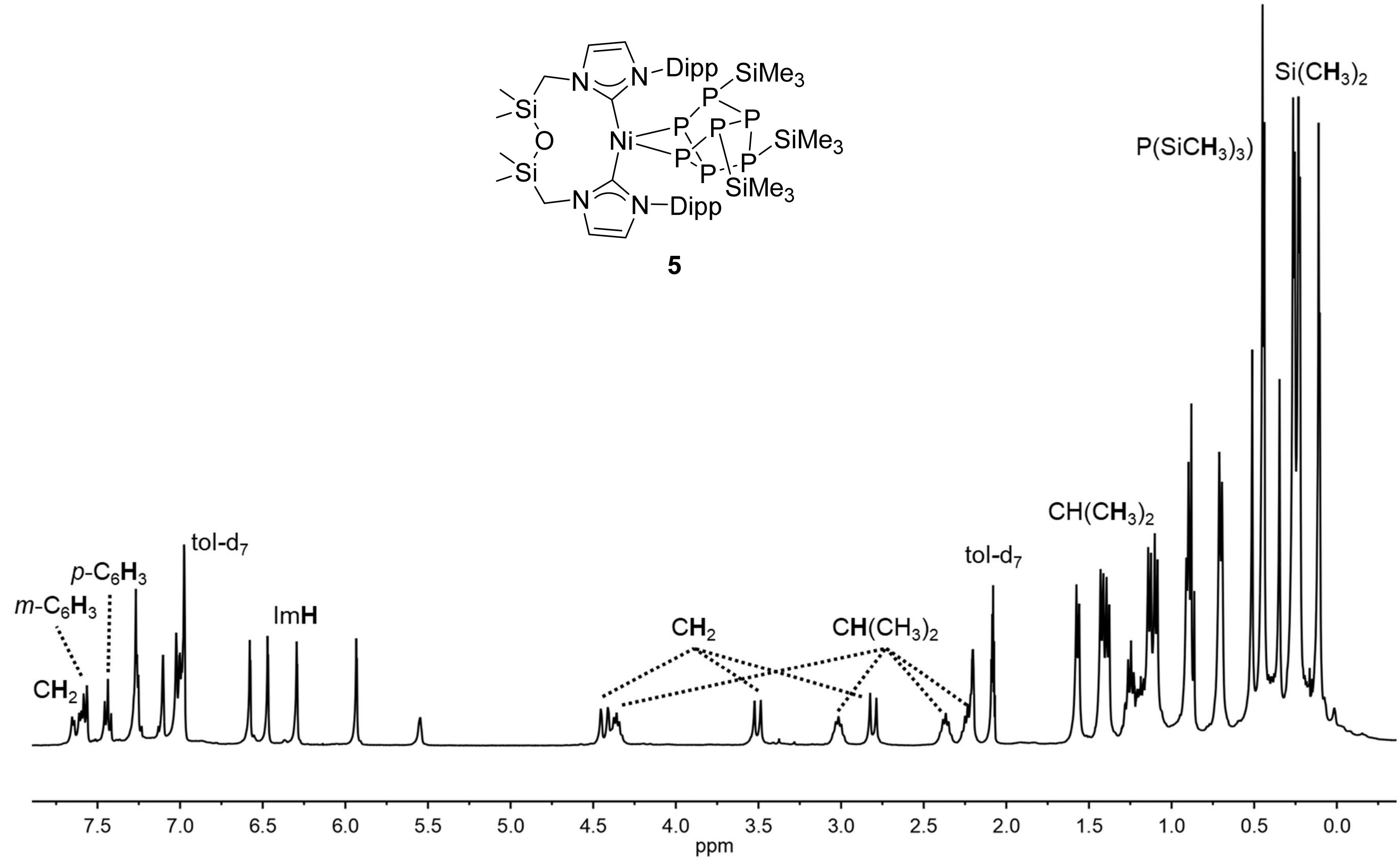

Figure S13. ^{1}H NMR spectrum of **5** (400 MHz, toluene-d_8, 278 K).

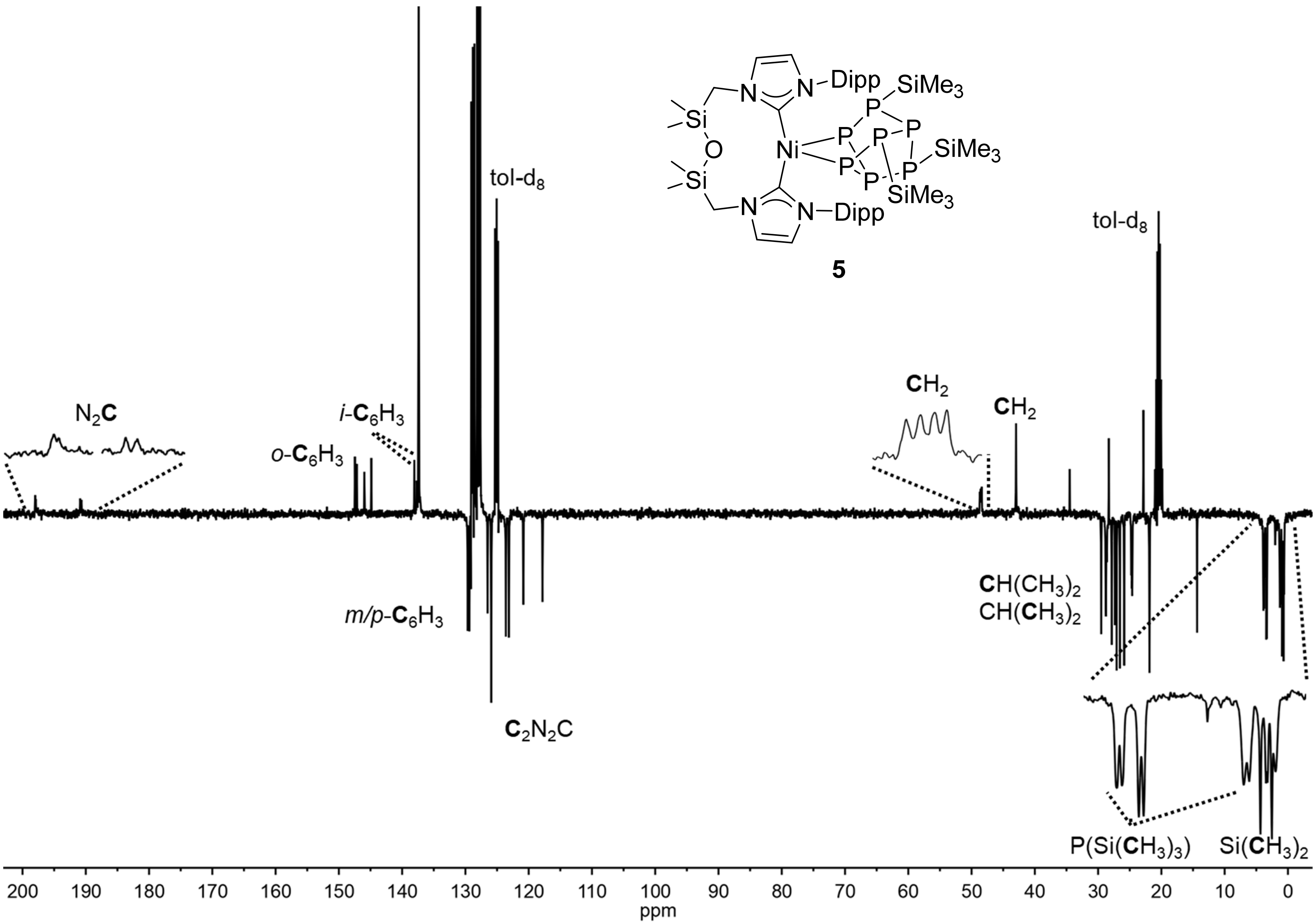

Figure S14. ^{13}C DEPTQ NMR spectrum of **5** (400 MHz, toluene-d_8, 278 K).

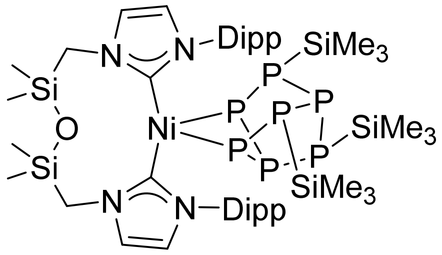

5

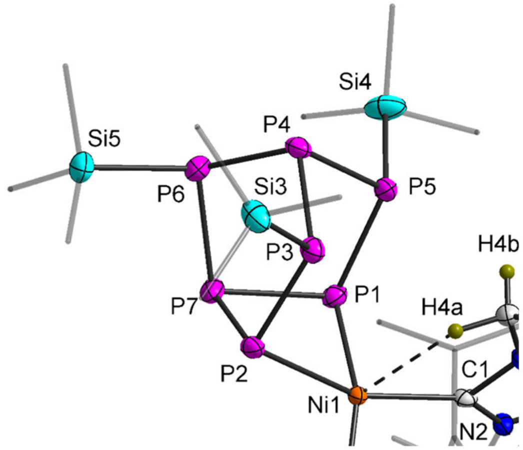

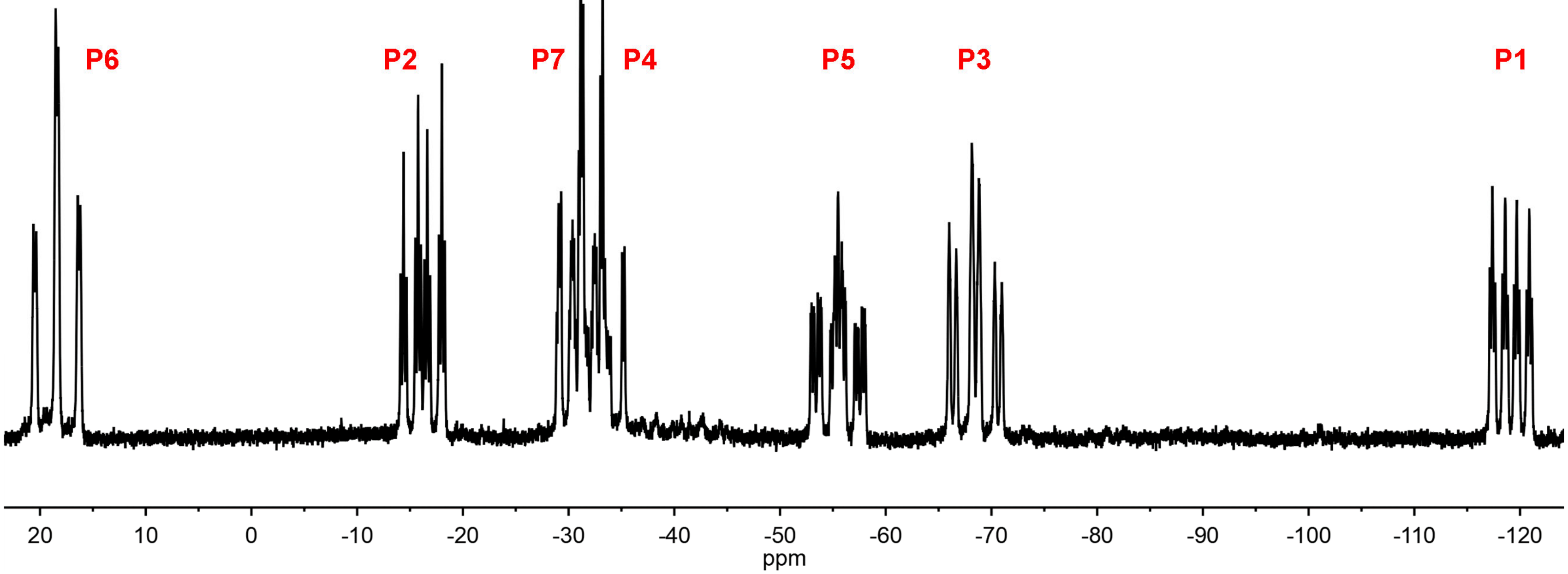

Figure S15. ^{31}P NMR spectrum of **5** (400 MHz, toluene-d_8, 278 K).

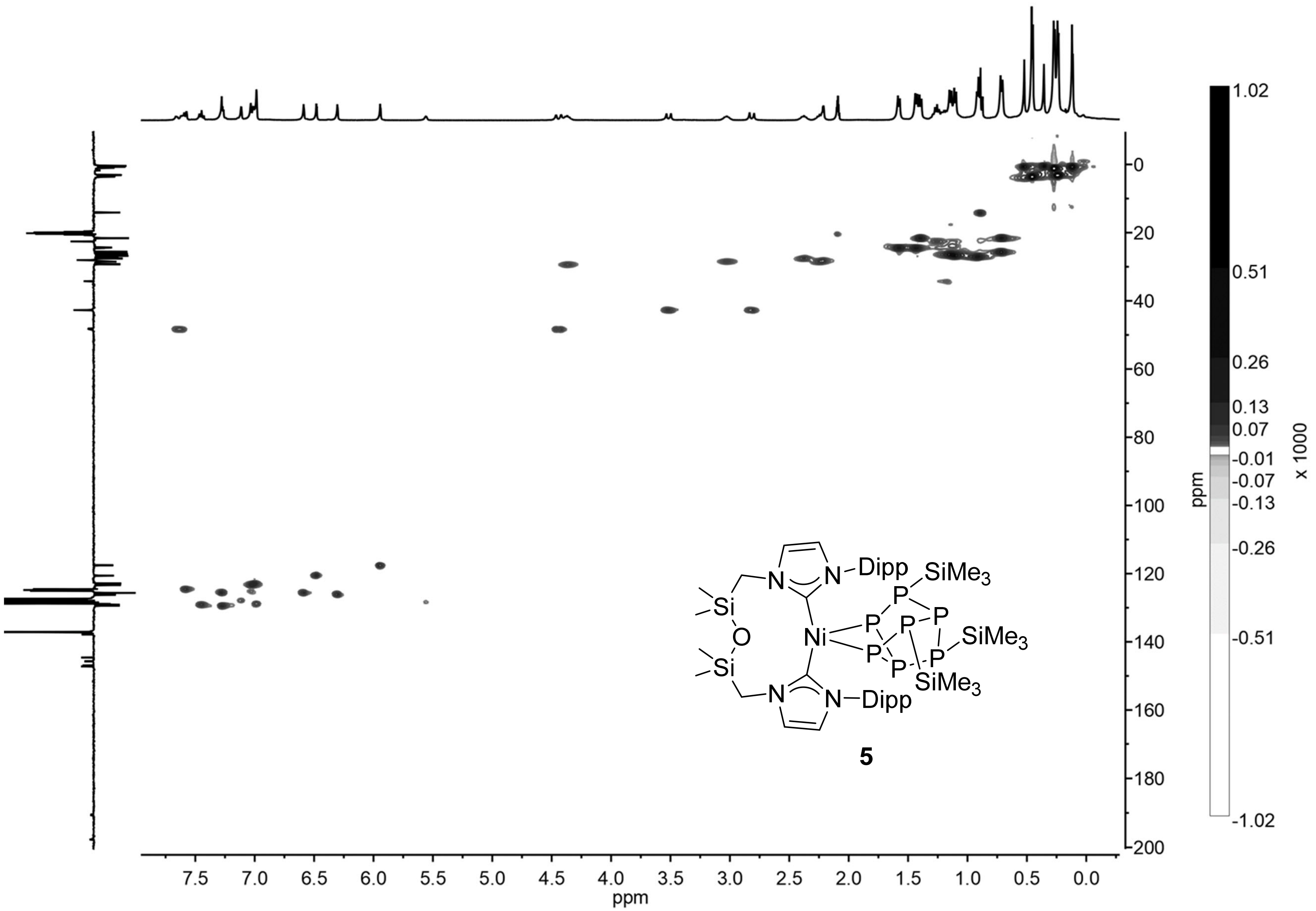

Figure S16. ^{1}H–^{13}C HSQC NMR spectrum of **5** (400 MHz, toluene-d_8, 278 K).

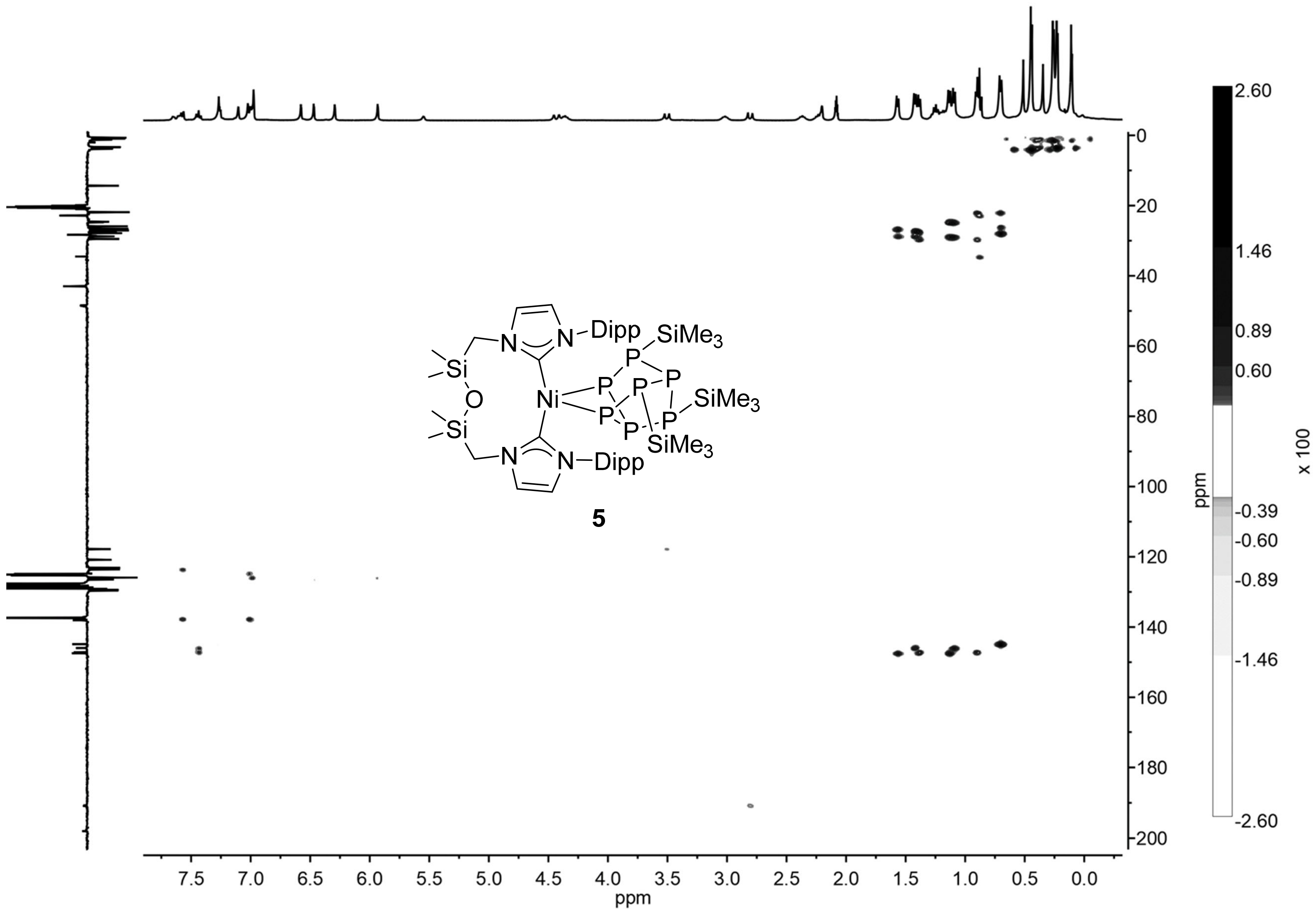

Figure S17. ^{1}H–^{13}C HMBC NMR spectrum of **5** (400 MHz, toluene-d_8, 278 K).

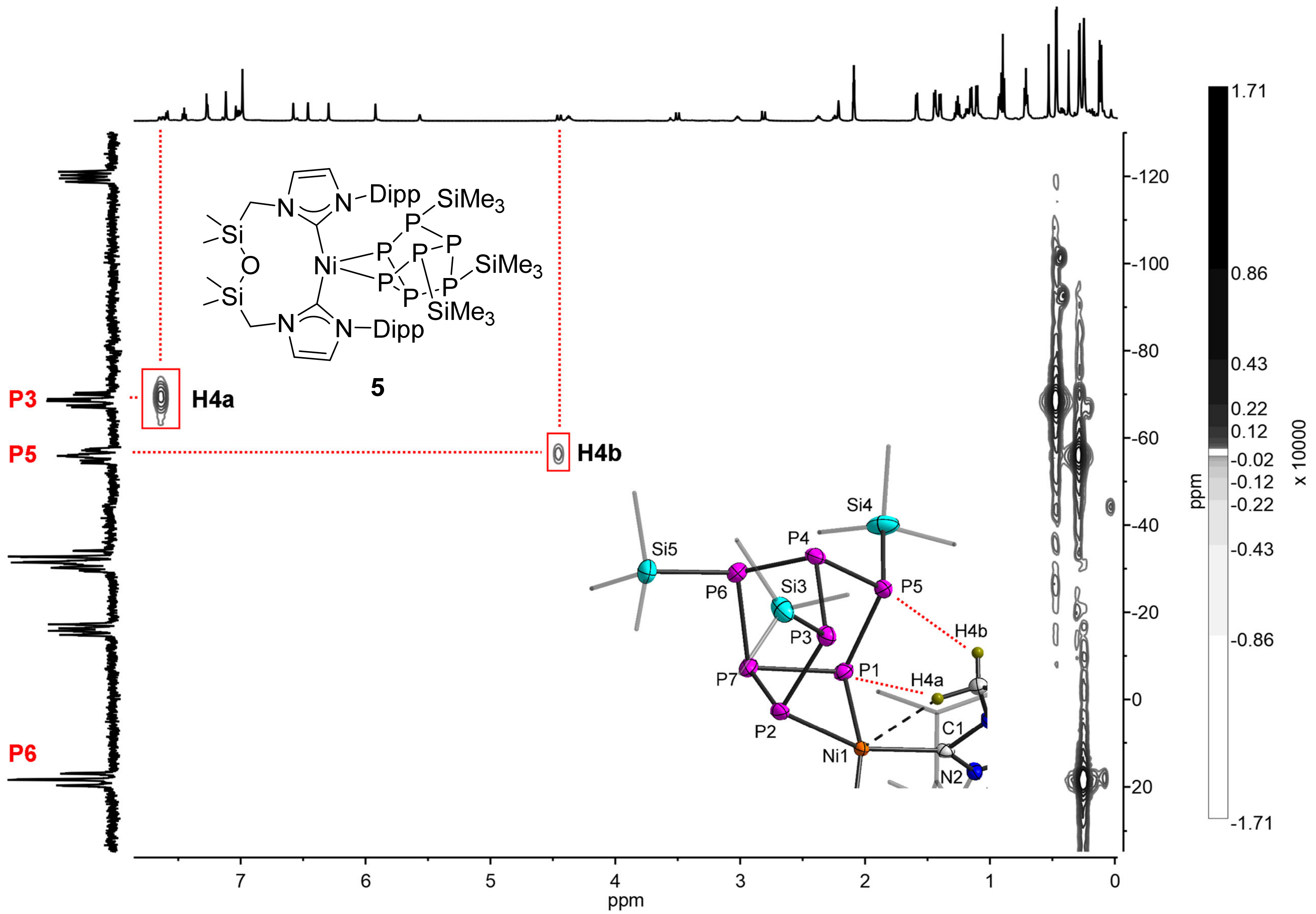

Figure S18. $^{1}H–^{31}P$ HSQC NMR spectrum of **5** (400 MHz, toluene-d_8, 278 K).

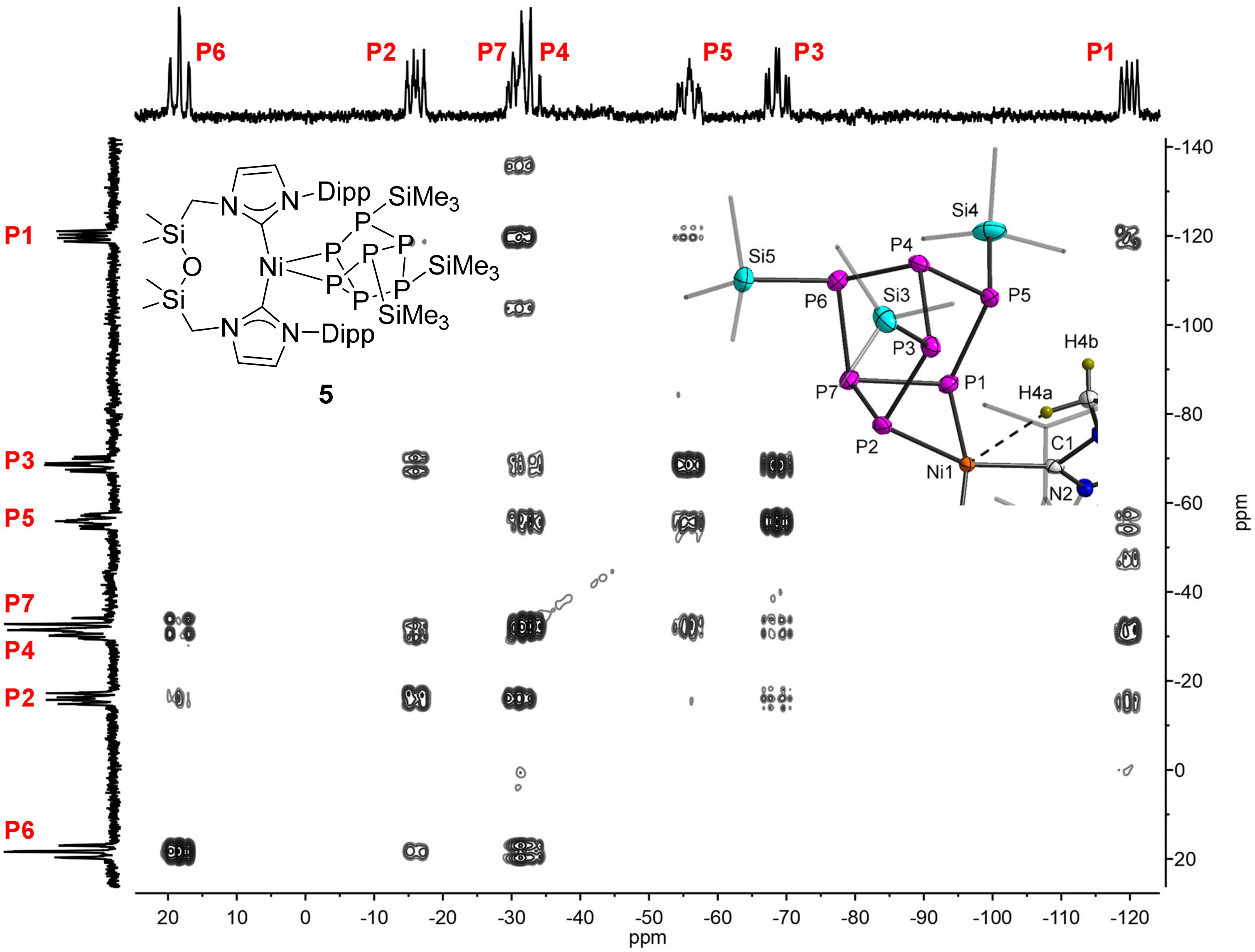

Figure S19. ^{31}P–^{31}P COSY NMR spectrum of **5** (400 MHz, toluene-d_8, 278 K).

Crystallographic Details

Table S1. Summary of Crystallographic Data for Compounds **3**–**5**.

	**3**	**4**	**5**
CCDC Deposition	2115737	2115738	2115739
Formula	$C_{72}H_{108}N_8Ni_2O_2P_4Si_4$	$C_{72}H_{108}N_8Ni_2O_2P_2Si_4$	$C_{45}H_{81}N_4NiOP_7Si_5$
Formula weight	1471.32	1409.38	1110.08
Crystal system	Triclinic	monoclinic	Orthorhombic
Space group	P −1	P 2_1/n	P b c a
a(Å)	15.1033(5)	13.0219(8)	16.0125(8)
b(Å)	15.1741(5)	44.536(3)	27.0370(14)
c(Å)	21.4008(8)	15.2665(11)	34.6364(16)
α(°)	106.010(2)	90	90
β(°)	93.243(2)	92.474(4)	90
γ(°)	106.507(2)	90	90
Volume ($Å^3$)	4471.4(3)	8845.4(10)	14995.1(13)
Z	2	4	8
Temp (K)	173(2)	173(2)	173(2)
λ, Å	0.71073	0.71073	0.71073
ρ(calc) gcm^{-1}	1.093	1.058	0.983
F(000)	1568	3016	4720
R(int)	0.1022	0.1017	0.1665
μ, mm^{-1}	0.588	0.557	0.516
θ range (°)	2.822–24.999	0.914–24.999	2.303–24.999
Total data	58513	80777	84248
Unique	15671	15521	13013
Completeness (%)	99.5	99.6	98.5
Parameters	869	835	601
R (>2σ)	0.0603	0.0640	0.0773
R_w (all data)	0.1485	0.1586	0.1696
GOF	0.998	1.045	1.039

Deposition numbers 2115737–2115739 contain the supplementary crystallographic data for this paper. These data are provided free of charge by the joint Cambridge Crystallographic Data Centre and Fachinformationszentrum Karlsruhe Access Structures service www.ccdc.cam.ac.uk/structures.

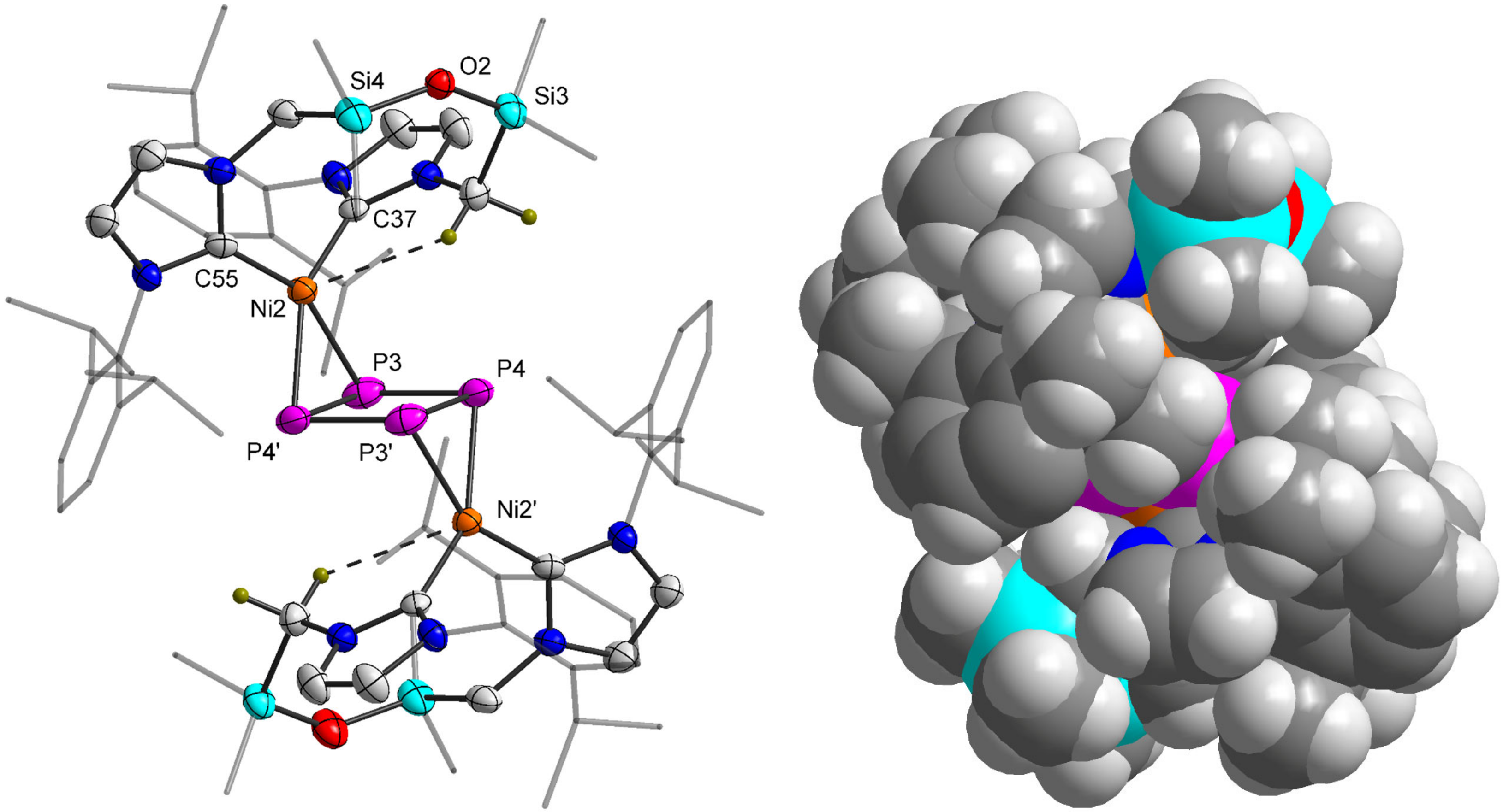

Figure S20. Structure of one of the two independent molecules of **3** in the solid state, with 50% probability thermal ellipsoids and hydrogen atoms not involved in anagostic interactions omitted for clarity (left) and space filling model of **3** (right). Selected bond distances [Å] and angles [º]: P3–P4' 2.145(2), 2.149(2), P3–P4 2.237(2), 2.242(2), Ni–P 2.2436(15)–2.2822(14), Ni–C 1.911(5)–1.933(5), Ni···H 2.74(5), 2.76(5), P–P–P 89.58(7) – 90.42(7), C–Ni–C 109.9(2), 110.8(2), P–Ni–P 56.58(5), 56.70(5), Si–O–Si 138.1(2), 139.8(2), NiP_2/P_4 96.64(5), 96.62(6), NiP_2/NiC_2 11.84(14), 17.90(11).

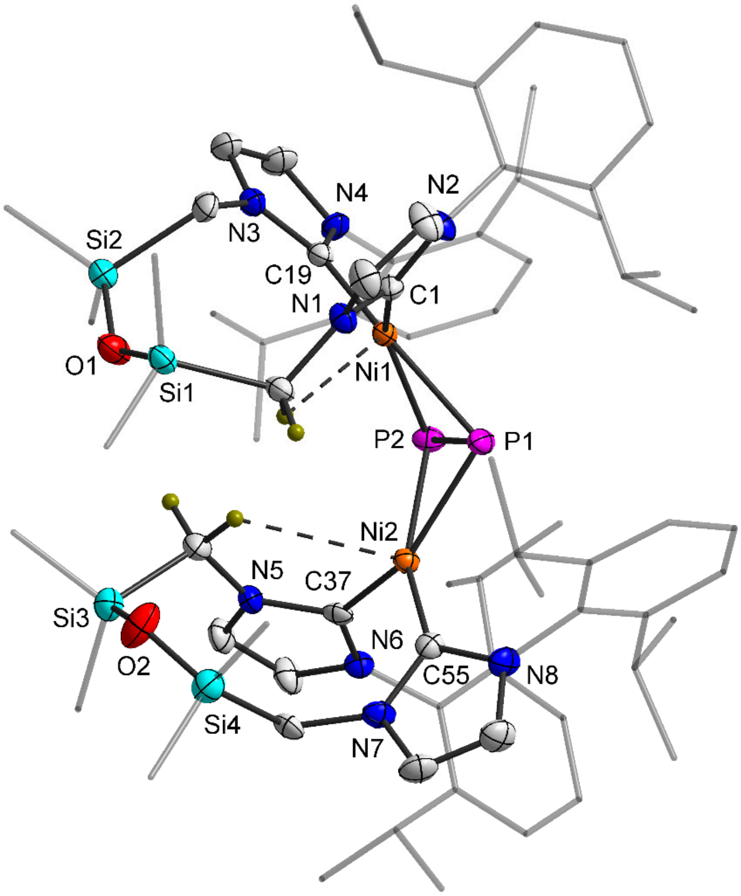

Figure S21. Molecular structure of **4** in the solid state, with 50% probability level thermal ellipsoids and hydrogen atoms not involved in anagostic interactions omitted for clarity. Selected bond distances [Å] and angles [º]: P–P 2.0784(16), Ni–P 2.2695(12)–2.2941(12), Ni–C 1.914(4)–1.938(4), Ni···H 2.75(3), 2.75(3), C–Ni–C 108.34(17), 108.39(17), P–Ni–P 54.17(4), 54.18(4), Si–O–Si 137.0(2), 140.6(2), NiP_2/NiP_2 120.87(3), NiP_2/NiC_2 9.0(1), 10.2(1).

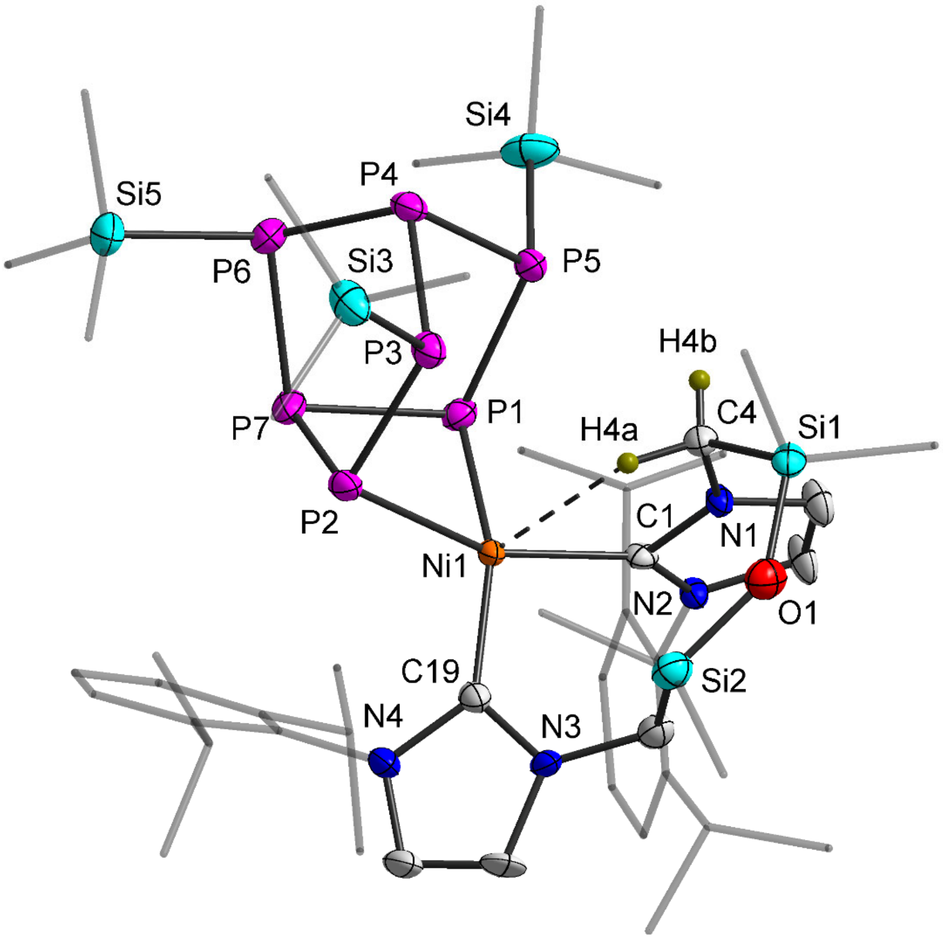

Figure S22. Molecular structure of **5** in the solid state, with 50% probability level thermal ellipsoids and hydrogen atoms not involved in anagostic interactions omitted for clarity. Selected bond distances [Å] and angles [º]: P4–P3 2.184(2), P4–P5 2.186(2), P4–P6 2.201(2), P1–P5 2.227(2), P2–P3 2.233(2), P7–P6 2.160(2), P1–P7 2.205(2), P2–P7 2.220(2), P1···P2 3.007(2), Ni–C 1.954(5), 1.992(5), Ni1–P1 2.2253(16), Ni1–P2 2.2950(16), Ni···H 2.51(6), C1–Ni1–C19 102.6(2), Si–O–Si 142.1(3), NiP_2/NiC_2 28.16(15).

Computational Details

General Considerations. Geometries of all studied systems were optimized in the gas phase with dispersion corrected density functional theory, namely the PBE1PBE functional,[4] def2-TZVP basis set,[5] and Grimme's D3 correction with Becke-Johnson damping,[6] using the Gaussian 16-C.01 program suite.[7] The structures were confirmed to be minima or transition states on the singlet potential energy hypersurface *via* calculation of the associated vibrational frequencies (all positive or one imaginary frequency, respectively). The Kohn-Sham determinants of optimized **3**, **3'**, **3''**, and **4** were tested for internal and external instabilities, and a restricted-unrestricted instability was detected for **3''**. Consequently, the structure of **3''** was re-optimized on the singlet potential energy surface using the same functional-basis set combination but employing the broken-symmetry formalism.

NMR Chemical Shifts. ^{31}P NMR shielding tensors were calculated for **3** (isotropic values 268.1 and 326.2 ppm), **3'** (274.7 and 335.0 ppm), and **4** (198.8 ppm) in their optimized geometries using the GIAO formalism[8] and the functional-basis set combination employed in geometry optimization. Setting the calculated isotropic value of **4** to match its experimental ^{31}P chemical shift of 144.7 ppm yields calculated chemical shifts of 17 and 75 ppm for **3** (experimental values 21 and 63 ppm, respectively) and 9 and 69 ppm for **3'**. The geometrical average of the calculated ^{31}P chemical shifts for **3** and **3'** is 44 ppm, which is an excellent match with the single broad resonance observed experimentally at around 45 ppm.

Bonding Analyses. Bonding in **3** and **4** was analyzed using the extended transition state (ETS) method for energy decomposition analysis (EDA) combined with the natural orbitals for chemical valence (NOCV) theory (ETS-NOCV)[9] as available in the ADF 2019.3 program package.[10] These calculations employed the PBE1PBE functional,[4] TZ2P STO-basis sets,[11] Grimme's D3 dispersion correction with Becke-Johnson damping,[6] and treatment of scalar relativistic effects using the zero-order regular-approximated (ZORA) Hamiltonian.[12] Singlet state uncharged fragments $((\mathbf{1})Ni)_2$ and P_4 and $((\mathbf{1})Ni)_2$ and P_2 were used to represent **3** and **4**, respectively.

The flow of charge between promolecular fragments $((\mathbf{1})Ni)_2$ and P_4/P_2 was quantified using Hirshfeld charge analysis[13] and Voronoi deformation densities,[14] which indicated a transfer of 0.80 and 0.72 e^-, respectively, from $((\mathbf{1})Ni)_2$ to P_4 in **3** and 0.55 and 0.52 e^-, respectively, from $((\mathbf{1})Ni)_2$

to P_2 in **4**. Energy decomposition analyses for **3** and **4** (in parenthesis) gave instantaneous interaction energies ΔE_{int} = −943 (−749) kJ mol^{-1} that can be split to contributions from Pauli repulsion, ΔE_{int} = 1835 (1641) kJ mol^{-1}, electrostatic interaction, ΔE_{int} = −1417 (−1285) kJ mol^{-1}, orbital interaction, ΔE_{int} = −1228 (−1027) kJ mol^{-1}, and dispersion, ΔE_{int} = −133 (−78) kJ mol^{-1}. The orbital interaction contributions can further be described using NOCVs and deformation densities shown in Figures S24 and S26. The donation from ((**1**)Ni)$_2$ to P_4/P_2 vastly outweighs the back-donation component in both cases.

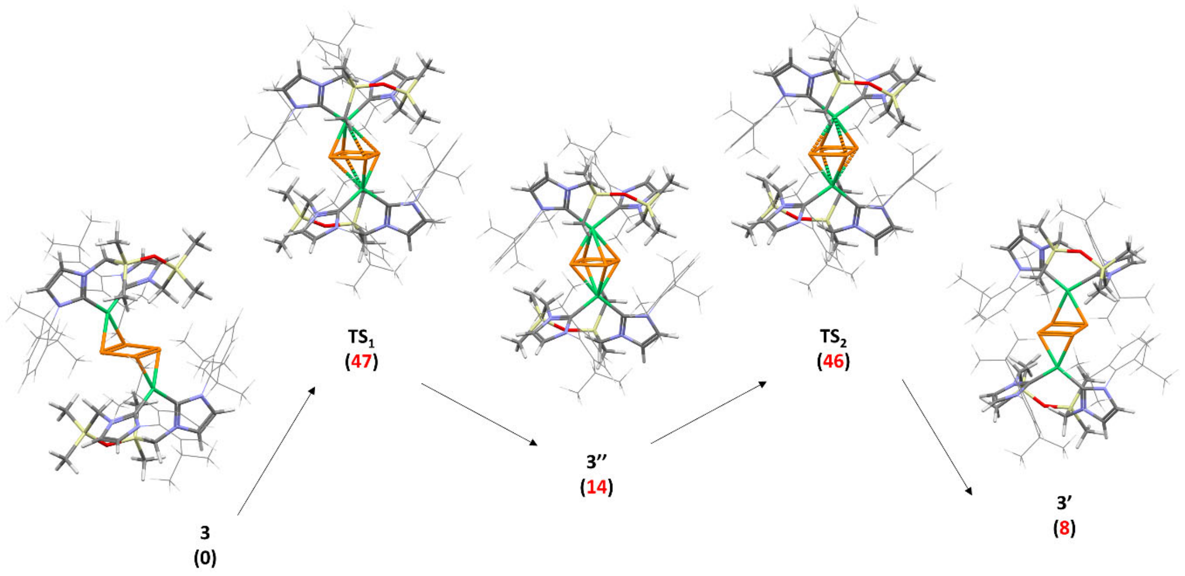

Figure S23. Calculated haptotropic rearrangement between **3** and **3'**, *via* **3''** over transition states **TS$_1$** and **TS$_2$**. Relative Gibbs free energies (in kJ mol^{-1} at 298 K) in parenthesis.

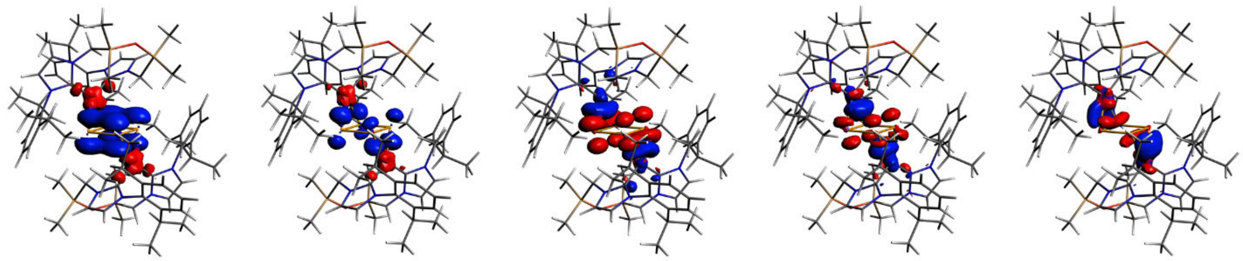

$\Delta\rho_1$ ΔE_{orb} = 576 | $\Delta\rho_2$ ΔE_{orb} = 367 | $\Delta\rho_3$ ΔE_{orb} = 62 | $\Delta\rho_4$ ΔE_{orb} = 49 | $\Delta\rho_5$ ΔE_{orb} = 44

Figure S24. Five most important ETS-NOCV deformation density contributions (isovalues = ±0.001–0.003 a.u.) to bonding between ((**1**)Ni)$_2$ and P_4 in **3**. Donation from ((**1**)Ni)$_2$ to P_4 ($\Delta\rho_1$ and $\Delta\rho_2$) and back-donation from P_4 to ((**1**)Ni)$_2$ ($\Delta\rho_3$–$\Delta\rho_5$). Contributions to the total orbital interaction term ΔE_{orb} are reported in kJ mol^{-1}. Red contour corresponds to depletion of electron density, whereas blue contour indicates accumulation of electron density.

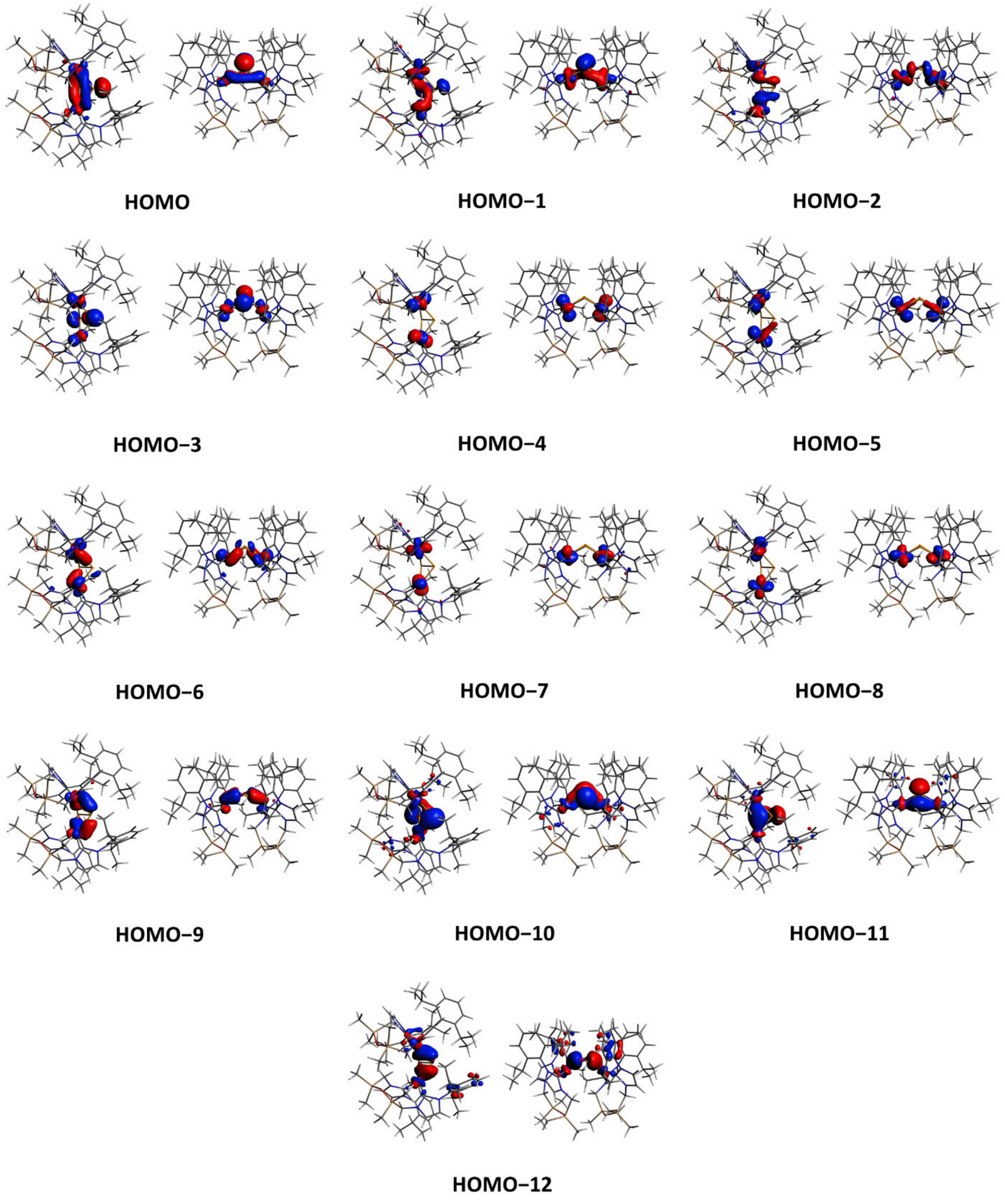

Figure S25. Frontier Kohn-Sham orbitals of **4** (isovalue = ±0.004 a.u.) in two different molecular orientations.

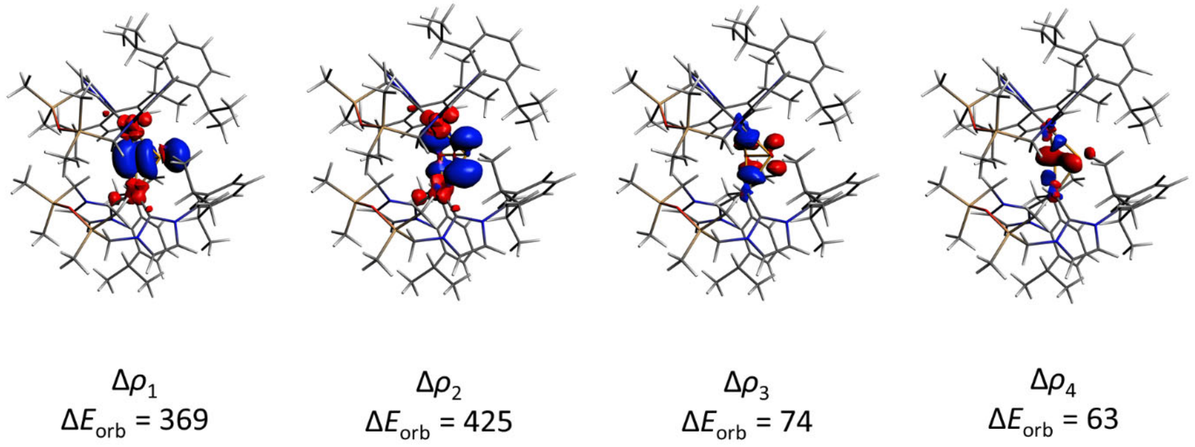

Figure S26. Four most important ETS-NOCV deformation density contributions (isovalues = ±0.001–0.003 a.u.) to bonding between ((**1**)Ni)$_2$ and P_2 in **4**. Donation from ((**1**)Ni)$_2$ to P_2 ($\Delta\rho_1$ and $\Delta\rho_2$) and back-donation from P_2 to ((**1**)Ni)$_2$ ($\Delta\rho_3$ and $\Delta\rho_4$). Contributions to the total orbital interaction term ΔE_{orb} are reported in kJ mol^{-1}. Red contour corresponds to depletion of electron density, whereas blue contour indicates accumulation of electron density.

References

[1] G. R. Fulmer, A. J. Miller, N. H. Sherden, H. E. Gottlieb, A. Nudelman, B. M. Stoltz, J. E. Bercaw, K. I. Goldberg, *Organometallics* **2010**, *29*, 2176–2179.

[2] For a wider selection of nickel complexes with substituent free P_x ligands, see: a) M. Di Vaira, C. A. Ghilardi, S. Midollini, L. Sacconi, *J. Am. Chem. Soc.* **1978**, *100*, 2550–2551; b) M. Di Vaira, S. Midollini, L. Sacconi, *J. Am. Chem. Soc.* **1979**, *101*, 1757–1763; c) O. J. Scherer, J. Braun, P. Walther, G. Wolmershäuser, *Chem. Ber.* **1992**, *125*, 2661–2665; d) V. Miluykov, A. Kataev, O. Sinyashin, P. Lönnecke, E. Hey-Hawkins, *Organometallics* **2005**, *24*, 2233–2236; e) E. Mädl, G. Balázs, E. V. Peresypkina, M. Scheer, *Angew. Chem. Int. Ed.* **2016**, *55*, 7702–7707; *Angew. Chem.* **2016**, *128*, 7833–7838; f) G. Hierlmeier, A. Hinz, R. Wolf, J. M. Goicoechea, *Angew. Chem. Int. Ed.* **2018**, *57*, 431–436; *Angew. Chem.* **2018**, *130*, 439–444; g) G. Hierlmeier, P. Coburger, N. P. van Leest, B. de Bruin, R. Wolf, *Angew. Chem. Int. Ed.* **2020**, *59*, 14148–14153; *Angew. Chem.* **2020**, *132*, 14252–14257.

[3] a) J.-C. Hierso, *Chem. Rev.* **2014**, *114*, 4838−4867; b) Y. Xiao, J. T. Mague, R. A. Pascal, Jr., *Eur. J. Org. Chem.* **2018**, 5706–5710.

[4] a) J. P. Perdew, K. Burke, M. Ernzerhof, *Phys. Rev. Lett.* **1996**, *77*, 3865–3868; b) J. P. Perdew, K. Burke, M. Ernzerhof, *Phys. Rev. Lett.* **1996**, *78*, 1396–1396; c) C. Adamo, V. Barone, *J. Chem. Phys.* **1999**, *110*, 6158–6170; d) M. Ernzerhof, G. E. Scuseria, *J. Chem. Phys.* **1999**, *110*, 5029–5036.

[5] F. Weigend, R. Ahlrichs, *Phys. Chem. Chem. Phys.* **2005**, *7*, 3297–3305.

[6] a) S. Grimme, J. Antony, S. Ehrlich, H. A. Krieg, *J. Chem. Phys.* **2010**, *132*, 154104/1–19 b) S. Grimme, S. Ehrlich, L. Goerigk, *J. Comput. Chem.* **2011**, *32*, 1456–1465.

[7] Gaussian 16, Revision C.01, M. J. Frisch, G. W. Trucks, H. B. Schlegel, G. E. Scuseria, M. A. Robb, J. R. Cheeseman, G. Scalmani, V. Barone, G. A. Petersson, H. Nakatsuji, X. Li, M. Caricato, A. V. Marenich, J. Bloino, B. G. Janesko, R. Gomperts, B. Mennucci, H. P. Hratchian, J. V. Ortiz, A. F. Izmaylov, J. L. Sonnenberg, D. Williams-Young, F. Ding, F. Lipparini, F. Egidi, J. Goings, B. Peng, A. Petrone, T. Henderson, D. Ranasinghe, V. G.

Zakrzewski, J. Gao, N. Rega, G. Zheng, W. Liang, M. Hada, M. Ehara, K. Toyota, R. Fukuda, J. Hasegawa, M. Ishida, T. Nakajima, Y. Honda, O. Kitao, H. Nakai, T. Vreven, K. Throssell, J. A. Montgomery, Jr., J. E. Peralta, F. Ogliaro, M. J. Bearpark, J. J. Heyd, E. N. Brothers, K. N. Kudin, V. N. Staroverov, T. A. Keith, R. Kobayashi, J. Normand, K. Raghavachari, A. P. Rendell, J. C. Burant, S. S. Iyengar, J. Tomasi, M. Cossi, J. M. Millam, M. Klene, C. Adamo, R. Cammi, J. W. Ochterski, R. L. Martin, K. Morokuma, O. Farkas, J. B. Foresman, and D. J. Fox, Gaussian, Inc., Wallingford CT, **2016**.

[8] K. Wolinski, J. F. Hilton, P. Pulay, *J. Am. Chem. Soc.* **1990**, *112*, 8251–8260.

[9] M. P. Mitoraj, A. Michalak, T. A. Ziegler, *J. Chem. Theory Comput.* **2009**, *5*, 962–975.

[10] a) G. te Velde, F. M. Bickelhaupt, E. J. Baerends, C. Fonseca Guerra, S. J. A. van Gisbergen, J. G. Snijders, T. Ziegler, *J. Comput. Chem.* **2001**, *22*, 931–967; b) ADF 2019.3, SCM, Theoretical Chemistry; Vrije Universiteit; Amsterdam; The Netherlands, http://www.scm.com.

[11] E. van Lenthe, E. J. Baerends, *J. Comput. Chem.* **2003**, *24*, 1142–1156.

[12] E. van Lenthe, E. J. Baerends, J. G. Snijders, *J. Chem. Phys.* **1994**, *101*, 9783–9792.

[13] F. L. Hirshfeld, *Theor. Chim. Acta*, **1977**, *44*, 129–138.

[14] C. Fonseca Guerra, J. W. Handgraaf, E. J. Baerends, F. M. Bickelhaupt, *J. Comput. Chem.* **2004**, *25*, 189–210.
